# Associations of Maternal Educational Level, Proximity to Green Space During Pregnancy, and Gestational Diabetes With Body Mass Index From Infancy to Early Adulthood: A Proof-of-Concept Federated Analysis in 18 Birth Cohorts

**DOI:** 10.1093/aje/kwad206

**Published:** 2023-10-19

**Authors:** Tim Cadman, Ahmed Elhakeem, Johan Lerbech Vinther, Demetris Avraam, Paula Carrasco, Lucinda Calas, Marloes Cardol, Marie-Aline Charles, Eva Corpeleijn, Sarah Crozier, Montserrat de Castro, Marisa Estarlich, Amanda Fernandes, Serena Fossatti, Dariusz Gruszfeld, Kathrin Guerlich, Veit Grote, Sido Haakma, Jennifer R Harris, Barbara Heude, Rae-Chi Huang, Jesús Ibarluzea, Hazel Inskip, Vincent Jaddoe, Berthold Koletzko, Sandrine Lioret, Veronica Luque, Yannis Manios, Giovenale Moirano, George Moschonis, Johanna Nader, Mark Nieuwenhuijsen, Anne-Marie Nybo Andersen, Rosie McEachen, Angela Pinot de Moira, Maja Popovic, Theano Roumeliotaki, Theodosia Salika, Loreto Santa Marina, Susana Santos, Sylvain Serbert, Evangelia Tzorovili, Marina Vafeiadi, Elvira Verduci, Martine Vrijheid, T G M Vrijkotte, Marieke Welten, John Wright, Tiffany C Yang, Daniela Zugna, Deborah Lawlor

**Keywords:** BMI, cohort data sharing, federated analyses, gestational diabetes, green spaces, maternal education

## Abstract

International sharing of cohort data for research is important and challenging. We explored the feasibility of multicohort federated analyses by examining associations between 3 pregnancy exposures (maternal education, exposure to green vegetation, and gestational diabetes) and offspring body mass index (BMI) from infancy to age 17 years. We used data from 18 cohorts (*n* = 206,180 mother-child pairs) from the EU Child Cohort Network and derived BMI at ages 0–1, 2–3, 4–7, 8–13, and 14–17 years. Associations were estimated using linear regression via 1-stage individual participant data meta-analysis using DataSHIELD. Associations between lower maternal education and higher child BMI emerged from age 4 and increased with age (difference in BMI *z* score comparing low with high education, at age 2–3 years = 0.03 (95% confidence interval (CI): 0.00, 0.05), at 4–7 years = 0.16 (95% CI: 0.14, 0.17), and at 8–13 years = 0.24 (95% CI: 0.22, 0.26)). Gestational diabetes was positively associated with BMI from age 8 years (BMI *z* score difference = 0.18, 95% CI: 0.12, 0.25) but not at younger ages; however, associations attenuated towards the null when restricted to cohorts that measured gestational diabetes via universal screening. Exposure to green vegetation was weakly associated with higher BMI up to age 1 year but not at older ages. Opportunities of cross-cohort federated analyses are discussed.

## Abbreviations

BMI: body mass index
ECCN: EU Child Cohort Network
GDM: gestational diabetes mellitus
IPD: individual participant data
SEP: socioeconomic position

Prospective cohort studies contribute to important research questions, but they are resource intensive. Over recent decades, international funders and cohort data custodians have emphasized the importance of data sharing (1–4). This provides economic efficiency, enables replication and triangulation of findings across different studies, increases the period of the life course that can be studied for repeated measures, and increases statistical power particularly for rare outcomes.

To meet this challenge, the EU Child Cohort Network (ECCN) has been created to address key research questions about the associations of early life stressors with health from infancy to adulthood (5, 6). The ECCN is an open and sustainable network of 17 birth cohorts across 12 countries in Europe and Australia comprising more than 250,000 participants. In addition to increasing power and supporting replication, this network contains extensive repeated-measured data and thus enables researchers to explore how associations might differ across the life course.

The aim of this paper is to use the ECCN to explore the feasibility of multicohort federated analyses (“federated” describes the analysis of multiple data sets) by examining associations between different pregnancy exposures and offspring body mass index (BMI) from infancy to 17 years. BMI was chosen as a suitable outcome for this proof-of-concept study, as reducing childhood overweight and obesity is a major global public health challenge, and it is hypothesized that higher BMI starts to be “programmed” in intrauterine and early infancy (7, 8). Furthermore, as weight and height are often measured at repeated time points, it provides an opportunity to investigate potential changes in exposure-BMI associations at different ages across the life course.

We chose 3 pregnancy exposures that were hypothesized to influence offspring BMI and that would be useful to illustrate different challenges in federated analyses (e.g., harmonization and missingness): 1) maternal education, 2) exposure to green vegetation, and 3) gestational diabetes (GDM). We chose maternal education as an example of a categorical variable with low levels of missing data in ECCN and for which there is extensive previous research on associations with BMI. We chose exposure to green vegetation as a continuous, area-based variable, with high levels of missing data due to some cohorts in ECCN not having geographic data. Finally, we chose GDM as a categorical variable harmonized from diverse sources of information (e.g., retrospective self-report, health record extraction and diagnosis made on the basis of results from blood samples). The exposures are briefly summarized below.

Maternal education: Socioeconomic position (SEP) is a complex exposure encompassing several different domains of family resources with maternal education at birth a commonly used indicator. Lower maternal education birth is associated with higher child BMI in medium- and high-income countries (9–14). SEP likely influences childhood BMI through exposure to an obesogenic environment (15–17). While studies have consistently found lower family SEP to be associated with higher child BMI, evidence regarding the age at which these inequalities emerge and the course they take is not consistent. (9–13, 18, 19).

Residential proximity to green space: Maternal availability of green spaces could influence offspring BMI via increased physical activity during pregnancy, stress reduction, or reduced exposure to pollution (20, 21). Some studies have reported that higher postnatal exposure to green spaces is associated with lower BMI, but evidence is not conclusive (22–24). While increased prenatal exposure to green spaces has been consistently associated with higher birth weight (25, 26), little is known about associations with BMI at older ages (27).

Gestational diabetes mellitus (GDM, defined as hyperglycemia in pregnancy) (28) is robustly associated with higher mean birth weight and being large for gestational age (29–32). Higher birth weight is in turn associated with higher future offspring BMI, fat mass, and lean mass (33, 34); thus, it has been proposed that intrauterine fetal overgrowth related to higher maternal circulating glucose may result in lifelong higher offspring BMI (35, 36). However, few studies have explored whether any association of GDM with offspring BMI changes as the offspring age. This is important as a lasting effect into older age and/or an increasing effect across both childhood and adulthood might lead to higher risk of adverse adult cardiometabolic outcomes than association limited only to childhood (37).

The aims of this study, therefore, were to explore the feasibility of multicohort federated analyses by examining associations between 3 pregnancy exposures (maternal education, green spaces, and GDM) and BMI measured at 5 age periods across childhood. We hypothesized that those whose parents had lower educational attainment and those exposed in utero to maternal gestational diabetes would have higher BMI. As evidence for the association of maternal gestational access to green space and offspring BMI is limited, we had no specific hypothesis for the association.

## METHODS

### Inclusion criteria and participating cohorts

**Figure 1:**
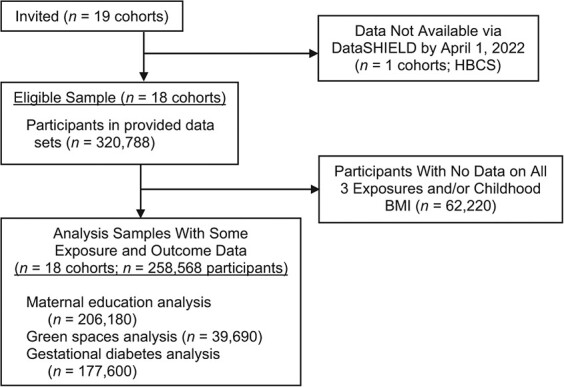
Flow chart of cohorts and participants for a study of associations of maternal educational level, proximity to green space during pregnancy, and gestational diabetes with body mass index (BMI) from infancy to early adulthood, multiple countries. Signed agreements received and DataSHIELD access credentials provided. HBCS, Helsinki Birth Cohort Study.

Pregnancy and birth cohort studies from the ECCN were eligible to participate if they: 1) had information on at least one of the 4 exposures and BMI measured at a minimum of 1 time point; 2) the study was approved by their institutional review boards; and 3) the infrastructure for federated analysis was established. Further details of each cohort can be found in Jaddoe et al. (5) and each cohort’s profile paper. All 17 core cohorts were invited, plus 2 additional cohorts from the wider LifeCycle network (Web Appendix 1, available at https://doi.org/10.1093/aje/kwad206) that had harmonized data available (The Amsterdam Born Children and their Development cohort (ABCD) (38) and The Healthy Growth Study, (HGS) (39)). Of these 19 studies, 18 were able to participate (the Helsinki Birth Cohort Study was unable to participate as they had not implemented the required infrastructure; Table 1). The analysis sample thus consisted of these 18 cohorts, with a maximum sample size of *n* = 206,180 (Figure 1). All participants gave written informed consent and ethical approval was granted by local ethics boards (Web Appendix 2). The analysis plan can be viewed at https://osf.io/58vau/.

**Table 1:** Summary of Cohort Characteristics, EU Child Cohort Network

**Cohort Name^a^**	**Country**	**City/Area**	**Design**	**No. of Included Children**	**Birth Year**	**Age Range of Included Children, years**
ABCD	Netherlands	Amsterdam	Prospective	6,152	2003–2004	0–12
ALSPAC	United Kingdom	Greater Bristol	Prospective	10,499	1991–1992	0–17
BiB	United Kingdom	Bradford	Prospective	13,400	2007–2011	0–10
CHOP	Germany, Belgium, Italy, Poland, Spain	Munich, Nuremberg, Liege, Brussels, Milano, Warsaw, Reus, Tarragona	Prospective	1,669	2002–2004	0–11
DNBC	Denmark	Greater Copenhagen	Prospective	77,534	1996–2002	0–18
EDEN	France	Nancy and Poitiers	Prospective	1,765	2003–2005	0–12
ELFE	France		Prospective	17,926	2011	0–9
GECKO	Netherlands	Drenthe	Prospective	2,748	2006–2007	0–11
GENR	Netherlands	Rotterdam	Prospective	8,680	2002–2006	0–10
HGS	Greece	Attica, Etoloakarnania, Thessaloniki, Iraklion	Cross-sectional^b^	2,570	1994–2000	10–14
INMA	Spain	Gipuzkoa, Sabadell, Valencia	Prospective	1,918	2003–2008	0–11
MoBa	Norway		Prospective	85,589	1999–2008	0–13
NFBC66	Finland		Prospective	7,709	1966	0–16
NFBC86	Finland		Prospective	7,315	1985–1986	0–16
NINFEA	Italy	Florence, Rome, Turin	Prospective	6,532	2005–2016	0–14
Raine	Australia	Perth	Prospective	2,548	1989–1992	1–17
Rhea	Greece	Crete	Prospective	1,002	2007–2008	0–12
SWS	United Kingdom	Southampton	Prospective	3,012	1998–2007	0–10

Abbreviations: ABCD, Amsterdam Born Children and their Development; ALSPAC, Avon Longitudinal Study of Parents and Children; BIB, Born in Bradford; CHOP, The EU Childhood Obesity Programme; DNBC, Danish National Birth Cohort; EDEN, Study on the Pre- and Early Postnatal Determinants of Child Health and Development; ELFE, Etude Longitudinale Francaise depuis l'Enfance; GECKO, Groningen Expert Center for Kids with Obesity Drenthe Cohort; GenR, Generation R; HGS, Healthy Growth Study; INMA, NMA-Infancia y Medio Ambiente (Environment and Childhood Project); MoBa, Norwegian Mother, Father, and Child Cohort Study; NFBC66, Northern Finland Birth Cohort 1966; NFBC86, Northern Finland Birth Cohort 1986; NINFEA, Nascita e INFanzia: gli Effetti dell'Ambiente; Raine, The Raine Study; Rhea, Mother Child Cohort in Crete; SWS, Southampton Women's Survey.

a ^a^ Descriptions of all the cohorts can be found in Web Appendix 2.

b ^b^ Information on early life exposures collected retrospectively.

### Exposures

#### Maternal education at birth.

A harmonized maternal education variable was created in each cohort based on the International Standard Classification of Education 97 (ISCED-97) and consisted of 3 categories: low (no education to lower secondary; ISCED-97 categories 0–2), medium (upper and postsecondary; ISCED-97 categories 3–4), high (degree and above; ISCED-97 categories 5–6) (40). Data was available in all cohorts.

#### Green spaces.

Exposure to green space during pregnancy was captured using normalized difference vegetation index (NDVI) within a 300-m buffer from the residential address using geographic information systems approaches previously described (41). NDVI (range 0–1) quantifies vegetation by measuring the difference between near-infrared and red-light reflection based on satellite imagery. Extremely low values (0–0.1) indicate areas of barren rock, sand, or snow; moderate values (0.2–0.5) indicate sparse vegetation such as shrubs and grasslands, and high values (0.6–1) indicate dense vegetation (42). Data was available for 9 cohorts (ALSPAC, BiB, DNBC, EDEN, GENR, INMA, MoBa, NINFEA, and Rhea; all the study names are defined in Table 1, and descriptions of all the cohorts can be found in Web Appendix 2.).

#### Gestational diabetes.

A binary variable indicating the presence or absence of evidence for GDM was harmonized for each cohort based either on extraction from clinical records or maternal self-report (Web Table 1). Data was available for all cohorts except HGS, NFBC66, and NFBC86. For most of the included cohorts at the time of pregnancy, no universal diagnostic test was used, meaning that selective misclassification of some women with GDM being treated as “healthy,” particularly if they had no clear risk factors for GDM, is possible. To test this, we performed a sensitivity analysis using data from only those studies in which all women in the sample had a blood measure of hyperglycemia, including glycated hemoglobin (HbA1c), fasting or random glucose, or oral glucose challenge or tolerance test (BiB and EDEN).

### Outcome

The outcome was offspring BMI *z* scores based on either clinical or parent-reported height and weight measurements (Web Table 2). Sex- and age-specific *z* scores were calculated per month for BMI using external World Health Organization standards (43) and references (44) excluding observations of 5 standard deviations or more from the population median. Separate BMI *z* scores were calculated for 5 age periods defined a priori: 1) 0–1 years, 2) 2–3 years, 3) 4–7 years, 4) 8–13 years, and 5) 14–17 years. These represent key developmental periods of change (early infancy, preschool, adiposity rebound, puberty, and late adolescence). Only 1 measurement per child was included within each period; therefore, if children had more than 1 measurement within an age bracket we used the earliest. A summary of the number of observations provided by each child is provided in Web Table 3.

### Confounders

We defined confounders as any factor that plausibly causes the exposure and offspring BMI, and we used directed acyclic graphs to depict these and determine whether there was any evidence of colliders that we should not adjust for (Web Figure 1). All confounders were assessed via self-report except prepregnancy BMI, which was based on either self-report or clinical measurements of weight and height. For analyses of maternal education with offspring BMI, no confounders were included as we did not identify plausible causes of variation in both maternal education and offspring BMI. For analyses with NDVI as the exposure, we adjusted for maternal education, area deprivation, and parity. For analyses with GDM as the exposure, we adjusted for maternal education, maternal age at birth (years), maternal prepregnancy BMI (calculated as weight (kg)/ height (m)^2^), parity (nulliparous, multiparous), and maternal smoking during pregnancy (yes/no). In addition, all analyses were adjusted for cohort, child sex, and age at weight and height measurements (months) to improve statistical precision. All cohorts had some available data on the above confounders. Maternal ethnicity also fit our definition of a confounder for all exposures but was only available (defined as Western vs. other) in 8 of the 17 cohorts (ABCD, ALSPAC, BiB, ELFE, GECKO, GENR, INMA, and Raine; maximum *n* = 45,601, representing 22% of the 206,180 participants). In a sensitivity analyses we repeated all analyses in this subset of cohorts with additional adjustment for ethnicity.

### Federated analyses using DataSHIELD

All analyses were performed using DataSHIELD, version 6.1.0 (45), and R, version 3.5.2 (R Foundation for Statistical Computing, Vienna, Austria). Briefly, each participating cohort stored their harmonized data on a local server protected by a firewall. Researchers granted permission to access the data use DataSHIELD to conduct remote analysis of individual participant data. DataSHIELD provides data security by preventing researchers viewing, copying, or transferring any data. Instead, analysis commands are performed on each server, and only nondisclosive summary statistics are returned to the researcher.

The functionality available within DataSHIELD is continually being developed; however, at the time of writing, mixed effects models were not available. Therefore, associations between each exposure and BMI at each age period were tested using linear regression and 1-stage individual participant data (IPD) meta-analysis. For each exposure, we fitted 5 separate regression models where the outcome was child BMI *z* score calculated within each age period as described above. All regression models included a dummy variable for cohort and adjusted for confounders as described above. To explore potential selection bias (due to each cohort contributing different data at different ages), we repeated all analyses, restricting the sample to the subgroup of participants with data at the oldest age. We also repeated analyses using 2-stage IPD random effects meta-analysis to describe estimates within each cohort and explore between-cohort heterogeneity. We also assessed the influence of 2 large cohorts (DNBC and MoBa) by repeating analyses with these cohorts excluded.

### Missing data

The analysis sample consisted of participants with available data on at least 1 exposure and BMI in at least 1 outcome period. There were minimal differences between participants in the analysis sample and those who were excluded, except included participants had slightly lower education and had lower rates of smoking in pregnancy (Web Table 4). Multiple imputation was also not available within DataSHIELD; therefore, missing data were handled through complete-case analysis, with the percentage of participants who were complete cases ranging from 6% to 65% of eligible participants from all cohorts combined; the proportion with complete data decreased with increasing age (Web Table 5). In addition to attrition across all cohorts, one reason for the low percentage of complete cases in the oldest age bracket was that for one of the largest cohorts (DNBC) only a small percentage of children had reached this age at the time of analysis. Estimates from linear regression models using complete cases are unbiased if the chance of being missing is not associated with the outcome after adjusting for covariates (46). To explore this assumption, for each exposure-outcome analysis we derived a variable indicating whether each participant had complete data. We then regressed this variable on child BMI, adjusting for nonmissing covariates (Web Figure 2). For all exposures, associations between BMI at all ages and the chance of having complete data were close to null.

## RESULTS

### Participant characteristics

**Figure 2:**
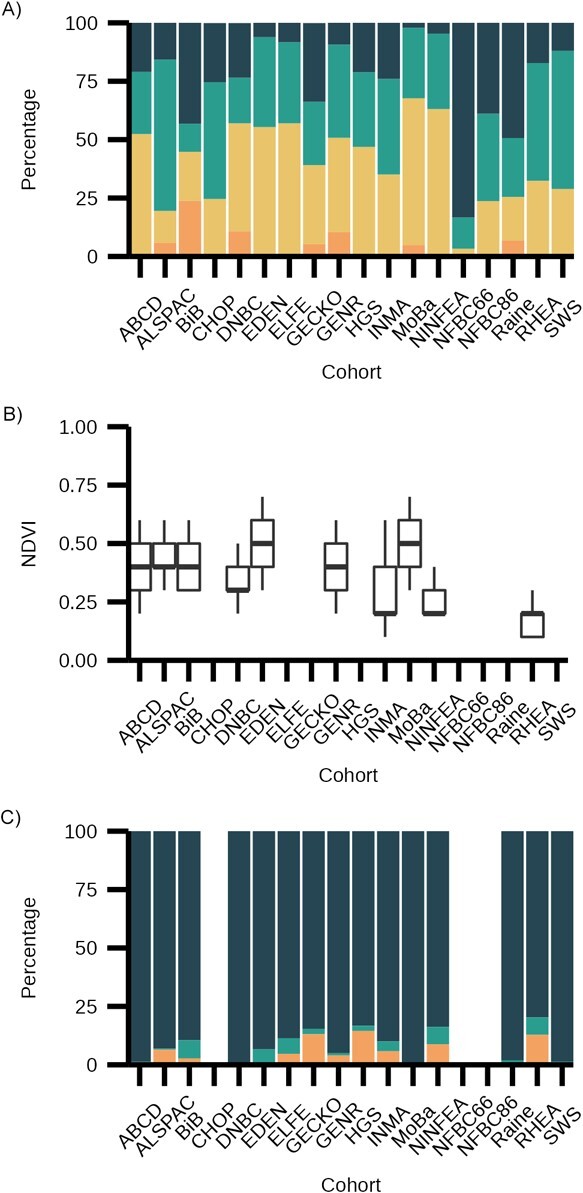
Exposure descriptive statistics for a study of associations of maternal educational level, proximity to green space during pregnancy, and gestational diabetes with body mass index (BMI) from infancy to early adulthood, multiple countries. A) Maternal education: dark blue = low education, teal = medium education, yellow = high education, orange = missing. B) Normalized difference vegetation index (NDVI). C) Gestational diabetes: dark blue = no pregnancy diabetes, teal = pregnancy diabetes, orange = missing. No data was available on gestational diabetes for the EU Childhood Obesity Programme (CHOP) as this was an exclusion criterion for entry into the study. For all other studies, figures are blank where the exposure is entirely missing. Values for NDVI represent median and interquartile range. All the study names are defined in Table 1, and descriptions of all the cohorts can be found in Web Appendix 2. Data was included from cohorts containing births from years 1966--2016.

The number of participants included in analyses ranged from 206,180 (maternal education and BMI for ages up to 1 year) to 7,096 (NDVI and BMI at ages 14–17 years). There were large differences between cohorts in the educational level of mothers, with MoBa and NINFEA containing mostly highly educated mothers while BiB, NFBC66, and Raine contained mothers with lowest levels of education (Figure 2A). INMA, NINFEA, and Rhea had the lowest values for NDVI, indicating exposure to lower levels of vegetation (Figure 2B). There was marked heterogeneity between cohorts in estimated rates of GDM (e.g., GENR = 0.8%, NINFEA = 8.1%; Figure 2C). Cohort-specific information on covariates and child BMI, height, weight, and age at measurement are shown in Web Tables 6–10.

### Associations between pregnancy exposures and child BMI

**Figure 3:**
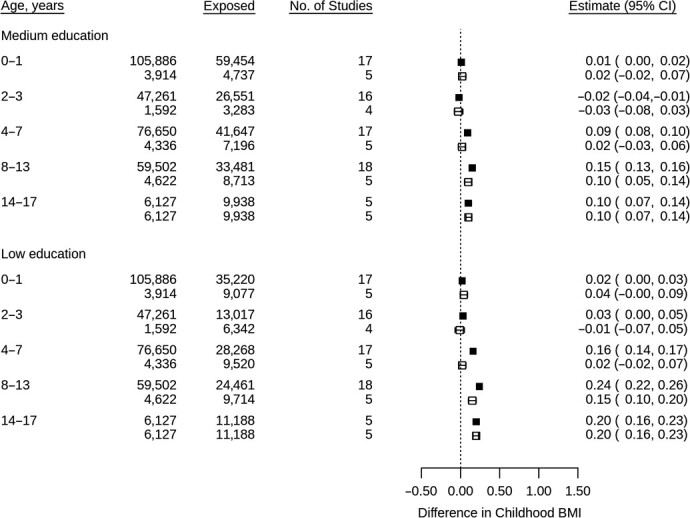
Associations between maternal education at birth and child body mass index *z* scores using 1-stage individual patient data meta-analysis of data from multiple countries, from cohorts containing births from years 1966--2016. Models adjusted for cohort, child sex, and exact age at measurement in days. Solid fill = maximum available sample; no fill = restricted to sample with available data at ages 14–17 years. BMI, body mass index.

**Figure 4:**
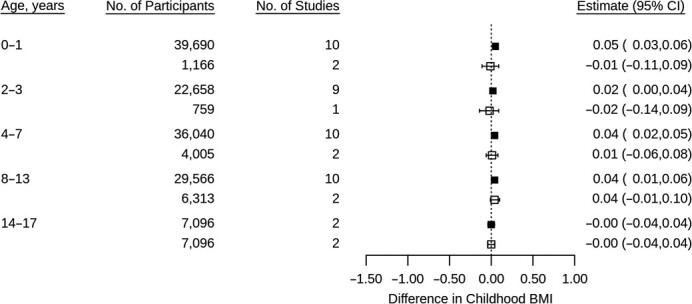
Associations between normalized difference vegetation index in pregnancy and child body mass index *z* scores using 1-stage individual patient data meta-analysis of data from multiple countries, from cohorts containing births from years 1966--2016. Models adjusted for cohort, child sex, exact age at measurement, maternal education, parity, and area deprivation. Normalized difference vegetation index scaled by interquartile range. Solid fill = maximum available sample; no fill = restricted to sample with available data at ages 14–17 years. BMI, body mass index.

**Figure 5:**
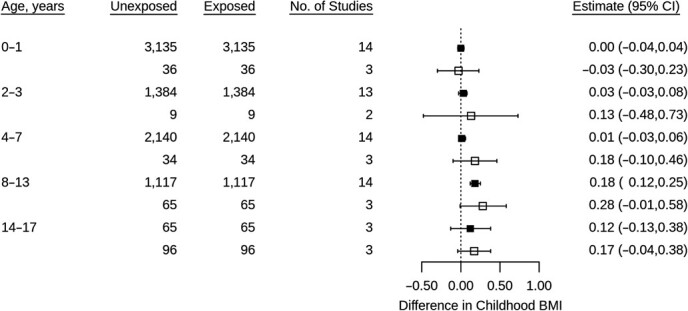
Associations between gestational diabetes and child body mass index *z* scores using 1-stage individual patient data meta-analysis of data from multiple countries, from cohorts containing births from years 1966--2016. Models adjusted for cohort, child sex, exact age at measurement, maternal education, maternal age at birth, prepregnancy body mass index (BMI), pregnancy smoking, and parity. Solid fill = maximum available sample; no fill = restricted to sample with available data at ages 14–17.

Figures 3–5 show associations between each exposure and BMI *z* scores within each age period. At ages 0–1 and 2–3 years, associations between maternal education and BMI were close to null; however, at older ages a consistent pattern emerged, with lower maternal education associated with higher childhood BMI (Figure 3). There was some evidence of a linear relationship as the magnitude of the association increased across categories of maternal education. Associations at earlier ages were slightly weaker when restricting analyses to the subgroup of participants with data at the oldest age. Results were similar using 2-stage IPD (Web Figures 3A–B), and while there was considerable heterogeneity between cohorts (*I*^2^ range, 0%–92%) the direction of association was largely consistent.

At ages 0–1, higher NDVI in pregnancy was associated with slightly higher BMI; however, at older ages, associations were close to null (Figure 4). Repeating analyses using 2-stage IPD showed considerable heterogeneity between cohorts (*I*^2^ range 0%–66%): For example at ages 2–3, higher NDVI was associated with higher BMI in BiB but lower BMI in Rhea (Web Figure 4).

Between ages 0 and 7, associations between GDM and childhood BMI were close to null; however, at ages 8–13, GDM was associated with higher BMI (Figure 5). Associations at all ages, except 0–1, were stronger when restricting to the subgroup of participants with data at the oldest age. These results were replicated using 2-stage IPD, with 10 out of 13 cohorts showing a positive association at ages 8–13 (*I*^2^ = 0–78%; Web Figure 5). At ages 14–17, associations attenuated towards null; however, within this age period only 3 cohorts had available data.

### Sensitivity and subgroup analyses

To test for potential confounding by indication, we repeated analyses for GDM comparing cohorts where assessment was via a universal blood-based glucose test versus self-report or nonuniversal test, and we found estimates to be lower for the 2 cohorts that used a universal blood-based test (Web Table 11). We additionally adjusted for ethnicity in the subset of up to 45,601 participants with available data, which attenuated associations of maternal education towards the null (Web Table 12). Finally we repeated analyses removing the 2 largest cohorts (DNBC and MoBa; Web Table 13); however, this did not change the direction of any associations or markedly change their magnitude.

## DISCUSSION

In this IPD meta-analysis of 18 cohort studies, with a maximum sample of 206,180 children, we explored the feasibility and utility of multicohort federated analysis by examining associations between key pregnancy exposures and BMI across childhood. We found consistent evidence that lower maternal education was associated with increased childhood BMI. Replicating and extending previous research (10–13, 18), we found this association to emerge from ages 4 to 7 years and increase in magnitude with age. Consistent with previous studies showing a positive association between exposure to green vegetation and birthweight (26, 27, 48, 49), we found that higher NDVI was associated with slightly higher BMI in the first year of life, although associations at older ages were close to null. We also found evidence that GDM was associated with higher child BMI at ages 8–13 but not at younger ages.

### Opportunities and challenges of federated data analysis

#### Data harmonization.

In the ECCN (5), we were interested in average associations across studies that were largely from different European countries, with a small number of studies from other high-income countries (e.g., Australia). As such, a substantial effort was made to harmonize data across all contributing studies (5, 6). Data harmonization is also commonly undertaken in nonfederated analyses; therefore, the opportunities and challenges discussed herein will have relevance to other analysis attempting to estimate average associations across studies using consistent data.

Individual participant pooling of studies (whether federated or not) provides the opportunity to increase statistical power and obtain more precise estimates than any single cohort, and to explore robustness of associations by examining consistency (replication) across independent populations. However, harmonization of data assessed in different ways and to different levels of detail can result in between-study heterogeneity that could complicate interpretation of results. For example, if different studies have used different methods for a binary variable, there may be different levels of misclassification between studies that are disguised by the harmonized binary variable.

As noted in the methods and results above, this was a concern for the harmonized GDM variable. For most studies, this was assessed via self-report, in populations where policy-dictated diagnostic tests were only done in those with risk factors at the first antenatal clinic visit, which could introduce confounding by indication. To explore this, we undertook a sensitivity analysis comparing pooled results from the 2 studies that had diagnosed GDM in all women using a blood-based measure of circulating glucose to those from remaining studies. The finding of a weaker association in those with the universal blood test suggests that our concerns regarding confounding by indication may be valid. While the harmonized binary variable can contain all data from all of the studies, we would recommend that other pooled individual participant studies undertake similar subgroup analyses where different methods have been used to assess a harmonized variable.

A further challenge with data harmonization is the loss of information through having to harmonize to the study with least detail for each measure, which could increase the risk of residual confounding. For example, in the present study, maternal education was harmonized into 3 categories from the more granular detail available within many of the cohorts, and similarly maternal smoking in pregnancy was harmonized to yes versus no, when several studies had more detailed measures on amount and timing of smoking (e.g., if a woman had smoked periconceptually and quit before pregnancy). Thus, the associations of GDM with offspring BMI might be influenced by residual confounding due to this “lowest common denominator.” SEP might be expected to confound away from the null, but as smoking results in lower BMI (47), it might mask an association (confound towards the null). As there are many different measures across the studies for both maternal education and smoking, to explore the possible effect of this in subgroups would result in groups with small numbers, for which robustly identifying between subgroup heterogeneity would be difficult and counter one of the key benefits of larger sample sizes.

#### Available analytical methods.

A key opportunity of federated analysis is the ability to analyze data from multiple cohorts without the need for data transfer. This minimizes the administrative burden of data transfer agreements and governance issues related to physical data sharing. In contrast to the traditional approach (where researchers from separate institutions run analyses which are then meta-analyzed by a central group), the federated approach is more time efficient and flexible as one researcher can perform all analyses and combine results. However, a limitation with DataSHIELD is that only a small subset of R packages are available so far, as any new packages need to be integrated and tested to ensure that disclosure risk is minimized.

While many R packages are now implemented in DataSHIELD (e.g., Metafor (48)), at the time of analysis, 2 methods were not available: multiple imputation and mixed effects models. In the absence of multiple imputation we used complete-case analysis, which in some scenarios carries the risk of bias (47). To explore potential selection bias due to attrition and cohorts differentially contributing data to different analyses, we repeated analyses restricting to the subgroup of participants with data at the oldest age period. For analyses with maternal education as the exposure we found lower estimates in this subgroup at earlier ages which may suggest potential selection bias with results at the older age being underestimated. By contrast, for analyses with gestational diabetes as the exposures we found stronger estimates at earlier ages suggesting that estimates at older ages may be overestimated. Where complete-case analysis is used, we therefore suggest that, at minimum, authors fully describe missing data, consider its likely mechanisms, and explore the potential for bias where possible.

The unavailability of mixed effects models meant that we were not able to explore associations with change in BMI as offspring aged in a way that accounted for correlation between repeat measures. Mixed effects models would also have enabled us to use all available data from participants with at least one measure of BMI, under a missing at random assumption (49, 50). Notwithstanding, our results for example with maternal education are broadly similar to models which did use trajectory analysis (i.e., showing widening inequalities) (12). DataSHIELD is a continually evolving project, and the implementation of other new methods is underway.

### Summary and future implications

In this multicohort study with 18 cohorts and up to 206,180 participants, we have illustrated potential scientific gains of collaboration and data sharing between international birth cohorts. We have demonstrated how federated analysis using DataSHIELD with cohorts from the ECCN provides opportunities to tackle research questions with increased statistical power and the ability to explore consistency (replication) across independent studies without the need to share data. We acknowledge and demonstrate the possibility of bias and residual confounding resulting from harmonizing data across multiple cohorts and the limitations that result from federated data platforms, i.e., not having more advanced data analysis methods. While we have focused here on DataSHIELD, we expect that other federated analysis platforms will similarly focus on straightforward descriptive and generalized regression models as more advanced methods are added to the platform.

## Supplementary Material

Web_Material_kwad206
