# Supplementary material for: Associations of Maternal Educational Level, Proximity to Green Space During Pregnancy, and Gestational Diabetes With Body Mass Index From Infancy to Early Adulthood: A Proof-of-Concept Federated Analysis in 18 Birth Cohorts
Source: Am J Epidemiol. 2023 Oct 19;193(5):753–63. doi: 10.1093/aje/kwad206 (PMC11367017; doi:10.1093/aje/kwad206)
Supplement: Web_Material_kwad206 [file web_material_kwad206.zip › kwad206 Cadman Web Material Final.pdf]

Web Material

Associations of Maternal Educational Level, Proximity to Greenspace During Pregnancy, and Gestational Diabetes With Body Mass Index From Infancy to Early Adulthood: A Proof-of-Concept Federated Analysis in 18 Birth Cohorts

Tim Cadman, Ahmed Elhakeem, Johan Lerbech Vinther, Demetris Avraam, Paula Carrasco, Lucinda Calas, Marloes Cardol, Marie-Aline Charles, Eva Corpeleijn, Sarah Crozier, Montserrat de Castro, Marisa Estarlich, Amanda Fernandes, Serena Fossatti, Dariusz Gruszfeld, Kathrin Gurlich, Veit Grote, Sido Haakma, Jennifer R. Harris, Barbara Heude, Rae-Chi Huang, Jesús Ibarluzea, Hazel Inskip, Vincent Jaddoe, Berthold Koletzko, Veronica Luque, Yannis Manios, Giovenale Moirano, George Moschonis, Johanna Nader, Mark Nieuwenhuijsen, Anne-Marie Nybo Andersen, Rosie McEachen, Angela Pinot de Moira, Maja Popovic, Theano Roumeliotaki, Theodosia Salika, Loreto Santa Marina, Susana Santos, Sylvain Serbert, Evangelia Tzorovili, Marina Vafeiadi, Elvira Verduci, Martine Vrijheid, TGM Vrijkotte, Marieke Welten, John Wright, Tiffany C Yang, Daniela Zugna, Deborah Lawlor

Table of Contents

WEB APPENDIX 1: THE LIFECYCLE PROJECT GROUP 3

WEB APPENDIX 2: STUDY SPECIFIC INFORMATION 5

WEB TABLE 1: INFORMATION SOURCES FOR GESTATIONAL DIABETES 11

WEB TABLE 2: COHORT-SPECIFIC METHODS OF DATA COLLECTION FOR HEIGHT AND WEIGHT 12

WEB TABLE 3: NUMBERS OF OBSERVATIONS PROVIDED BY EACH CHILD FOR EACH EXPOSURE 13

WEB TABLE 4: DESCRIPTIVE STATISTICS FOR ANALYSIS DATASET VS EXCLUDED PARTICIPANTS 14

WEB TABLE 5: NUMBERS OF COMPLETE CASES FOR EACH EXPOSURE-OUTCOME COMBINATION 15

WEB TABLE 6: COHORT-SPECIFIC INFORMATION ON COVARIATES 17

WEB TABLE 6: COHORT-SPECIFIC INFORMATION ON COVARIATES (CONTINUED) 18

WEB TABLE 7: CHILD BMI Z-SCORES BY COHORT 19

WEB TABLE 8: CHILD HEIGHT MEASUREMENTS (CM) BY COHORT 20

WEB TABLE 9: CHILD WEIGHT MEASUREMENTS (KG) BY COHORT 21

WEB TABLE 10: AGE AT HEIGHT AND WEIGHT MEASUREMENT (MONTHS) BY COHORT 22

WEB TABLE 11: GDM ANALYSIS STRATIFIED BY TEST TYPE 23

WEB TABLE 12: ANALYSIS ON SUBGROUP WITH ETHNICITY DATA 24

WEB TABLE 13: ANALYSES WITH DNBC & MOBA REMOVED 25

WEB FIGURE 1: DIRECTED ACYCLIC GRAPHS 26

WEB FIGURE 2: ASSOCIATIONS BETWEEN CHILD BMI Z-SCORES AND PROBABILITY OF BEING A COMPETE CASE 27

WEB FIGURE 3A: ASSOCIATIONS BETWEEN MATERNAL EDUCATION AND CHILD BMI Z-SCORES USING 2-STAGE IPD META-ANALYSIS (MEDIUM EDUCATION VS HIGH) 28

WEB FIGURE 3B: ASSOCIATIONS BETWEEN MATERNAL EDUCATION AND CHILD BMI Z-SCORES USING 2-STAGE IPD META-ANALYSIS (LOW EDUCATION VS HIGH) 29

WEB FIGURE 4: ASSOCIATIONS BETWEEN NORMALISED DIFFERENCE VEGETATION INDEX AND CHILD BMI Z-SCORES USING 2-STAGE IPD META-ANALYSIS 30

WEB FIGURE 5: ASSOCIATIONS BETWEEN GESTATIONAL DIABETES AND CHILD BMI Z-SCORES USING 2-STAGE IPD META-ANALYSIS 31

REFERENCES 32

Web appendix 1: The LifeCycle Project Group

Vincent W.V. Jaddoe[1,2], Janine F. Felix[1,2], Liesbeth Duijts[1,2], Hanan El Marroun[1,3,4], Romy Gaillard[1,2], Susana Santos[1,2], Madelon L. Geurtsen[1,2], Marjolein N. Kooijman[1,2], Sara M. Mensink-Bout[1,2], Florianne O.L. Vehmeijer[1,2], Ellis Voerman[1,2], Marieke Welten[1,2], Martine Vrijheid[5,6,7], Jordi Sunyer[5,6,7,8], Mark Nieuwenhuijsen[5,6,7], Xavier Basagaña[5,6,7], Mariona Bustamante[5,6,7], Maribel Casas[5,6,7], Montserrat de Castro[5,6,7], Lourdes Cirugeda[5,6,7], Sílvia Fernández-Barrés[5,6,7], Serena Fossati[5,6,7], Raquel Garcia[5,6,7], Mònica Guxens[3,5,6,7], Jordi Júlvez[5,6,9], Aitana Lertxundi[5,10,11], Nerea Lertxundi[10,11], Sabrina Llop[5,12], Mònica López-Vicente[2,3,6], Maria-Jose Lopez-Espinosa[5,12,13], Lea Maitre[6], Mario Murcia[12,14], Jose Urquiza[5,6,7], Charline Warembourg[5,6,7], Lorenzo Richiardi[15], Costanza Pizzi[15], Daniela Zugna[15], Maja Popovic[15], Elena Isaevska[15], Milena Maule[15], Chiara Moccia[15], Giovenale Moirano[15], Davide Rasella[15], Mark A Hanson[16,17], Hazel M. Inskip[17,18], Chandni Maria Jacob[16,17], Theodosia Salika[18], Deborah A. Lawlor[19,20,21], Ahmed Elhakeem[19,21], Tim Cadman[19,21], Anne-Marie Nybo Andersen[22], Angela Pinot de Moira[22], Katrine Strandberg-Larsen[22], Marie Pedersen[22], Johan L Vinther[22], John Wright[23], Rosemary R.C. McEachan[23], Paul Wilson[24], Dan Mason[23], Tiffany C. Yang[23], Morris A. Swertz[25,26], Eva Corpeleijn[27], Sido Haakma[25], Marloes Cardol[27], Esther van Enckevort[25,26], Eleanor Hyde[25,26], Salome Scholtens[25,26], Harold Snieder[27], Chris H.L. Thio[27], Marina Vafeiadi[28], Lida Chatzi[29], Katerina Margetaki[29], Theano Roumeliotaki[28], Jennifer R. Harris[31], Johanna L. Nader[32], Marie-Aline Charles[34,35], Barbara Heude[34], Lidia Panico[36], Mathieu Ichou[36], Blandine de Lauzon-Guillain[34], Patricia Dargent-Molina[34], Maxime Cornet[34], Sandra M. Florian[36], Faryal Harrar[34], Johanna Lepeule[37], Sandrine Lioret[34], Maria Melchior[38], Sabine Plancoulaine[34], Marjo-Riitta Järvelin[39,40,41,42], Sylvain Sebert[39], Minna Männikkö[43], Priyanka Parmar[39], Nina Rautio[39], Justiina Ronkainen[39], Mimmi Tolvanen[39], Johan G Eriksson[44,45,46,47], Tuija M. Mikkola[45,48], Berthold Koletzko[49], Veit Grote[49], Nicole Aumüller[49], Ricardo Closa-Monasterolo[50], Joaquin Escribano[50], Natalia Ferré[50], Dariusz Gruszfeld[51], Kathrin Gürlich[49], Jean-Paul Langhendries[52], Veronica Luque[50], Enrica Riva[53], Phillipp Schwarzfischer[49], Martina Totzauer[49], Elvira Verduci[53], Annick Xhonneux[52], Marta Zaragoza-Jordana[50], Maarten Lindeboom[54], Ameli Schwalber[55], Nina Donner[55], Rae-Chi Huang[56], Rachel E. Foong[56,57], Graham L. Hall[56,57], Ashleigh Lin[56], Jennie Carson[56], Phillip Melton[58,59], Sebastian Rauschert[56]

[1]Department of Pediatrics, Erasmus MC, University Medical Center Rotterdam, Rotterdam, the Netherlands. [2]The Generation R Study Group, Erasmus MC, University Medical Center Rotterdam, Rotterdam, the Netherlands. [3]Department of Child and Adolescent Psychiatry and Psychology, Erasmus MC, University Medical Center Rotterdam, Rotterdam, the Netherlands. [4]Department of Psychology, Education and Child Studies, Erasmus School of Social and Behavioural Sciences, Rotterdam, the Netherlands. [5]CIBER Epidemiología y Salud Pública (CIBERESP), Spain. [6]ISGlobal, Barcelona, Spain. [7]Universitat Pompeu Fabra (UPF), Barcelona, Spain. [8]IMIM (Hospital del Mar Medical Research Institute), Barcelona, Spain. [9]Institut d'Investigació Sanitària Pere Virgili (IISPV), Hospital Universitari Sant Joan de Reus, Reus, Spain. [10]Biodonostia, Health research institute, San Sebastian, Spain. [11]University of Basque Country (UPV/EHU), Spain. [12]Epidemiology and Environmental Health Joint Research Unit, FISABIO–Universitat Jaume I–Universitat de València, Valencia, Spain. [13]Faculty of Nursing and Chiropody, Universitat de València, Valencia, Spain. [14]Conselleria de Sanitat, Valencia, Spain. [15]Cancer Epidemiology Unit, Department of Medical Sciences, University of Turin, Turin, Italy. [16]Institute of Developmental Sciences, Faculty of Medicine, University of Southampton, Southampton, United Kingdom. [17]NIHR Southampton Biomedical Research Centre, University of Southampton and University Hospital Southampton NHS Foundation Trust, Southampton, United Kingdom. [18]MRC Lifecourse Epidemiology Centre, University of Southampton, Southampton General Hospital, Southampton, United Kingdom. [19]MRC Integrative Epidemiology Unit at the University of Bristol, Bristol, United Kingdom. [20]NIHR Bristol Biomedical Research Centre, Bristol, United Kingdom. [21]Population Health Science, Bristol Medical School, University of Bristol, Bristol, United Kingdom. [22]Section of Epidemiology, Department of Public Health, University of Copenhagen, Copenhagen, Denmark. [23]Bradford Institute for Health Research, Bradford Teaching Hospitals NHS Foundation Trust, Bradford, United Kingdom. [24]University of Manchester, Manchester, United Kingdom. [25]University of Groningen, University Medical Center Groningen, Genomics Coordination Center, Groningen, the Netherlands. [26]University of Groningen, University Medical Center Groningen, Department of Genetics, Groningen, the Netherlands. [27]Department of Epidemiology, University of Groningen, University Medical Center Groningen, Groningen, the Netherlands. [28]Department of Social Medicine, Faculty of Medicine, University of Crete, Heraklion, Crete, Greece. [29]Department of Preventive Medicine, Keck School of Medicine, University of Southern California, Los Angeles, CA, USA. [30]Centre for Fertility and Health, Norwegian Institute of Public Health, Oslo, Norway. [31]Division of Health Data and Digitalization, Norwegian Institute of Public Health, Oslo, Norway. [32]Department of Genetics and Bioinformatics, Division of Health Data and Digitalisation, Norwegian Institute of Public Health, Oslo, Norway. [33]Norwegian Institute of Public Health, Oslo, Norway.
Université de Paris Cité, Inserm, INRAE, Centre of Research in Epidemiology and StatisticS (CRESS), France. [35]ELFE Joint Unit, French Institute for Demographic Studies (INED), French Institute for Medical Research and H ealth (INSERM), French Blood Agency, Aubervilliers, France. [36]Institut National d'Etudes

Démographiques (INED), Aubervilliers, France. [37]Université Grenoble Alpes, Inserm, CNRS, Team of Environmental Epidemiology Applied to Reproduction and Respiratory Health, IAB, Grenoble, France. [38]Sorbonne Université, INSERM, Institut Pierre Louis d' Epidemiologie et de Santé Publique (IPLESP), Equipe de Recherche en Epidémiologie Sociale (ERES), Paris, France. [39]Center For Life-course Health research, Faculty of Medicine, University of Oulu, Oulu, Finland. [40]Department of Epidemiology and Biostatistics, MRC-PHE Centre for Environment and Health, School of Public Health, Imperial College London, London, United Kingdom. [41]Department of Life Sciences, College of Health and Life Sciences, Brunel University London, London, United Kingdom. [42]Unit of Primary Health Care, Oulu University Hospital, OYS, Oulu, Finland. [43]Infrastructure for Population Studies, Faculty of Medicine, University of Oulu, Oulu, Finland. [44]Department of General Practice and Primary Health Care, University of Helsinki and Helsinki University Hospital, Helsinki, Finland. [45]Folkhälsan Research Center, Helsinki, Finland. [46]Obstetrics & Gynecology, Yong Loo Lin School of Medicine, National University of Singapore and National University Health System, Singapore. [47]Singapore Institute for Clinical Sciences (SICS), Agency for Science and Technology (A*STAR), Singapore. [48]Clinicum, Faculty of Medicine, University of Helsinki, Helsinki, Finland. [49]Department of Pediatrics, Dr.von Hauner Children's Hospital, University Hospital, LMU, Munich, Germany. [50]Universitat Rovira i Virgili, IISPV, Tarragona, Spain. [51]Neonatal Department, Children's Memorial Health Institute, Warsaw, Poland. [52]CHC St Vincent, Liège-Rocourt, Belgium. [53]University of Milan, Milan, Italy. [54]Department of Economics, VU University Amsterdam, Amsterdam, the Netherlands. [55]Concentris Research Management GmbH, Fürstenfeldbruck, Germany. [56]Telethon Kids Institute, Perth, Western Australia, Australia. [57]School of Physiotherapy and Exercise Science, Curtin University, Perth, Western Australia, Australia. [58]Curtin/UWA Centre for Genetic Origins of Health and Disease, School of Biomedical Sciences, The University of Western Australia, Australia. [59]School of Pharmacy and Biomedical Sciences, Curtin University, Perth, Western Australia, Australia

Web appendix 2: Study specific information

ABCD

The Amsterdam Born Children and their Development cohort is a multi-ethnic birth cohort in the Netherlands focusing on maternal factors during pregnancy influencing offspring's health. Between January 2003 and March 2004 all pregnant women in Amsterdam (n=12,373) were invited for participation at their first pregnancy check-up at median 13 weeks' gestation. They were asked to fill out a pregnancy questionnaire. Of these 12,373 women, 8,266 filled out the pregnancy questionnaire (response 67 %) and 7050 granted permission for follow-up. More information can be found in the cohort profile [1].

The cohort was established with a significant funding from the Public Health Service and Municipal Council of Amsterdam. Additional funding was obtained from the Netherlands Organization for Health Research and Development (ZonMw), Amsterdam University Medical Center, Amsterdam, Dutch Heart Foundation and Sarphati Institute, Amsterdam.

Approval for the ABCD-study was obtained from the Central Committee on Research involving Human Subjects in the Netherlands, the Medical Ethical Committees of the participating hospitals, and from the Registration Committee of the Municipality of Amsterdam. Written informed consent was obtained from all participants.

The authors thank the participating mothers, fathers, their children, and all others who contributed to the ABCD-study: obstetric care providers, primary schools, students, and youth healthcare centers in Amsterdam, The Netherlands.

ALSPAC

Pregnant women resident in Avon, UK with expected dates of delivery 1st April 1991 to 31st December 1992 were invited to take part in the study. The initial number of pregnancies enrolled is 14,541 (for these at least one questionnaire has been returned or a "Children in Focus" clinic had been attended by 19/07/99). Of these initial pregnancies, there was a total of 14,676 foetuses, resulting in 14,062 live births and 13,988 children who were alive at 1 year of age.

When the oldest children were approximately 7 years of age, an attempt was made to bolster the initial sample with eligible cases who had failed to join the study originally. As a result, when considering variables collected from the age of seven onwards (and potentially abstracted from obstetric notes) there are data available for more than the 14,541 pregnancies mentioned above. The number of new pregnancies not in the initial sample (known as Phase I enrolment) that are currently represented on the built files and reflecting enrolment status at the age of 24 is 913 (456, 262 and 195 recruited during Phases II, III and IV respectively), resulting in an additional 913 children being enrolled. The phases of enrolment are described in more detail in the cohort profile paper and its update (see footnote 4 below). The total sample size for analyses using any data collected after the age of seven is therefore 15,454 pregnancies, resulting in 15,589 foetuses. Of these 14,901 were alive at 1 year of age.

A 10% sample of the ALSPAC cohort, known as the Children in Focus (CiF) group, attended clinics at the University of Bristol at various time intervals between 4 to 61 months of age. The CiF group were chosen at random from the last 6 months of ALSPAC births (1432 families attended at least one clinic). Excluded were those mothers who had moved out of the area or were lost to follow-up, and those partaking in another study of infant development in Avon.

Full details of the cohort can be provided in the cohort profiles [2, 3]. Please note that the study website contains details of all the data that is available through a fully searchable data dictionary and variable search tool: http://www.bristol.ac.uk/alspac/researchers/our-data/

The UK Medical Research Council and Wellcome (Grant ref: 217065/Z/19/Z) and the University of Bristol provide core support for ALSPAC. This publication is the work of the authors and Tim Cadman and Deborah Lawlor will serve as guarantors for the contents of this paper. A comprehensive list of grants funding is available on the ALSPAC website (http://www.bristol.ac.uk/alspac/external/documents/grant-acknowledgements.pdf); This research was specifically funded by H2020 LifeCycle project Grant Agreement No. 733206).

DAL and AK work in a unit that is supported by the University of Bristol and UK Medical Research Council (MC_UU_00011/6) and DAL holds a European Research Council Advanced Grant (ERC grant agreement no 669545) and is a NIHR Senior Investigator (NF-0616-10102). The funders had no role in the design of the study, the collection, analysis, or interpretation of the data; the writing of the manuscript, or the decision to submit the manuscript for publication. The views expressed in this paper are those of the authors and not necessarily those of any funder.

Ethical approval for the study was obtained from the ALSPAC Ethics and Law Committee and the Local Research Ethics Committees. Informed consent for the use of data collected via questionnaires and clinics was obtained from participants following the recommendations of the ALSPAC Ethics and Law Committee at the time.

We are extremely grateful to all of the families who took part in ALSPAC, the midwives for their help in recruiting them, and the whole ALSPAC team, which includes interviewers, computer and laboratory technicians, clerical workers, research scientists, volunteers, managers, receptionists and nurses.

BIB

BiB receives core infrastructure funding from the Wellcome Trust (WT101597MA) and a joint grant from the UK Medical Research Council (MRC) and Economic and Social Science Research Council (ESRC) (MR/N024397/1). This study has received support from the British Heart Foundation (CS/16/4/32482), US National Institutes of Health (R01 DK10324), European Research Council under the European Union's Seventh Framework Programme (FP7/2007-2013) / ERC grant agreement no 669545, and National Institute for Health Research ARC Yorkshire and Humber (NIHR200166. The views expressed are those of the author(s), and not necessarily those of the NHS, the NIHR or the Department of Health and Social Care.

Ethics approval has been obtained for the main platform study and all of the individual substudies from the Bradford Research Ethics Committee. All participants gave written informed consent.

The authors acknowledge that Born in Bradford is only possible because of the enthusiasm and commitment of the children and parents in Born in Bradford. We are grateful to all participants, health professionals and researchers who have made Born in Bradford happen.

CHOP

The CHOP study has been carried out with partial financial support from the Commission of the European Community, specific RTD Programme "Quality of Life and Management of Living Resources", within the Fifth Framework Program (research grants no. QLRT-2001-00389 and QLK1-CT-200230582), the Sixth Framework Program (contract no. 007036), and Seventh Framework Programme (EarlyNutrition; grant agreement no. 289346), the EU H2020 project LIFECYCLE under grant no. 733206 and the European Research Council Advanced Grant META-GROWTH (ERC-2012-AdG – no.322605) and with financial support from Polish Ministry of Science and Higher Education (2571/7.PR/2012/2). This manuscript does not necessarily reflect the views of the Commission and in no way anticipates the future policy in this area. No funding bodies had any role in the study design, data collection and analysis. Veronica Luque holds a Serra Hunter Fellowship.

The study was approved by the ethics committees of all study centers. Written informed parental consent was obtained for each infant.

The authors would particularly like to thank all the cohort participants for their generous collaboration. Furthermore, thanks to all persons who designed and conducted the study, entered the data, and participated in the data analysis and who are represented by the European Childhood Obesity Trial Study Group participants: B Koletzko, V Grote, M Totzauer, K Gürlich, P Schwarzfischer, N Aumüller, V Luque, M Zaragoza-Jordana, N Ferré, J Escribano, R Closa-Monasterolo, A Xhonneux, JP Langhendries, E Verduci, E Riva, D Gruszfeld.

DNBC

The Danish National Birth Cohort was established with a significant grant from the Danish National Research Foundation. Additional support was obtained from the Danish Regional Committees, the Pharmacy Foundation, the Egmont Foundation, the March of Dimes Birth Defects Foundation, the Health Foundation and other minor grants. The DNBC Biobank has been supported by the Novo Nordisk Foundation and the Lundbeck Foundation. Follow-up of mothers and children have been supported by the Danish Medical Research Council (SSVF 0646, 271-08-0839/06-066023, O602-01042B, 0602-02738B), the Lundbeck Foundation (195/04, R100-A9193), The Innovation Fund Denmark 0603-00294B (09-067124), the Nordea Foundation (02-2013-2014), Aarhus Ideas (AU R9-A959-13-S804), University of Copenhagen Strategic Grant (IFSV 2012), and the Danish Council for Independent Research (DFF – 4183-00594 and DFF - 4183-00152). AP is funded by a Lundbeck Foundation fellowship (R264-2017-3099).

The DNBC complies with the Declaration of Helsinki and was approved by the Danish National Committee on Biomedical Research Ethics. Informed consent was obtained from participants upon enrolment.

The authors would like to thank the participants, the first Principal Investigator of DNBC Prof. Jørn Olsen, the scientific managerial team, and DNBC secretariat for being, establishing, developing and consolidating the Danish National Birth Cohort.

EDEN

The EDEN study was supported by Foundation for medical research (FRM), National Agency for Research (ANR), National Institute for Research in Public health (IRESP: TGIR cohorte santé 2008 program), French Ministry of Health (DGS), French Ministry of Research, INSERM Bone and Joint Diseases National Research (PRO-A) and Human Nutrition National Research Programs, Paris-Sud University, Nestlé, French National Institute for Population Health Surveillance (InVS), French National Institute for Health Education (INPES), the European Union FP7 programmes (FP7/2007-2013, HELIX, ESCAPE, ENRIECO, Medall projects), Diabetes National Research Program (through a collaboration with the French Association of Diabetic Patients (AFD)), French Agency for Environmental Health Safety (now ANSES), Mutuelle Générale de l’Education Nationale a complementary health insurance (MGEN), French national agency for food security, French speaking association for the study of diabetes and metabolism (ALFEDIAM).

The study received approval from the ethics committee (CCPPRB) of Kremlin Bicêtre on 12 December 2002 and from CNIL (Commission Nationale Informatique et Liberté), the French data privacy institution. Women gave written informed consent for themselves and their child. Fathers gave written informed consent for themselves.

The authors thank the cohort participants and the EDEN mother-child study group, whose members are: I. Annesi-Maesano, J.Y. Bernard, J. Botton, M.A. Charles, P. Dargent-Molina, B. de Lauzon-Guillain, P. Ducimetière, M. de Agostini, B. Foliguet, A. Forhan, X. Fritel, A. Germa, V. Goua, R. Hankard, B. Heude, M. Kaminski, B. Larroque†, N. Lelong, J. Lepeule, G. Magnin, L. Marchand, C. Nabet, F Pierre, R. Slama, M.J. Saurel-Cubizolles, M. Schweitzer, O. Thiebaugeorges.

ELFE

The authors are grateful to 1) the former members of the Elfe unit without whom the project would never have started: Henri Léridon, initiator and former Principal Investigator of the project, Stéphanie Vandentorren, Claudine Pirus, and Ando Rakotonirina; 2) the expertise and assistance of members of the unit for support functions, 3) all the researchers who contribute to the projects as members of the Elfe thematic groups and especially their coordinators; 4) all the field research assistants and interviewers; 5) and above all, all the Elfe families who have placed their confidence in us and given up their time to the study.

Ethical approvals for data collection in maternity units and for each data collection wave during follow-up were obtained from the national advisory committee on information processing in health research (CCTIRS: Comité Consultatif sur le Traitement de l’Information en matière de Recherche dans le domaine de la Santé), the national data protection authority (CNIL: Comission Nationale Informatique et Liberté) and, in case of invasive data collection such as biological sampling, the committee for protection of persons engaged in research (CPP: Comité de Protection des Personnes). The ELFE study was also approved by the national committee for statistical

information (CNIS: Conseil National de l'Information Statistique). Informed consent was signed by the parents or the mother alone, with the father being informed of his right to deny consent for participation.

The Elfe cohort received funding from the National Research Agency Investment for the Future program [ANR-11-EQPX-0038]; French National Institute for Research in Public Health (IRESP TGIR 2009-2001 program); Ministry of Higher Education and Research; Ministry of Environment; Ministry of Health; French Agency for Public Health; Ministry of Culture; and National Family Allowance Fund.

GECKO Drenthe

The GECKO Drenthe birth cohort was funded by an unrestricted grant of Hutchison Whampoa Ld, Hong Kong and supported by the University of Groningen, Well Baby Clinic Foundation Icare, Noordlease, Paediatric Association Of The Netherlands and Youth Health Care Drenthe and the European Union's Horizon 2020 research and innovation programme (LIFECYCLE, grant agreement No 733206, 2016

This study was approved by the Medical Ethics Committee of the University Medical Center Groningen (UMCG). Parents of all participants in the study gave written informed consent.

The authors are grateful to the families who took part in the GECKO Drenthe study, the midwives, gyneacologists, nurses, and the general practitioners and all health professionals at the Preventive Child Healthcare Drenthe for their help in the recruitment and the measurements, and the GECKO Drenthe study team.

Generation R

The general design of the Generation R Study is made possible by financial support from the Erasmus MC, University Medical Center, Rotterdam, Erasmus University Rotterdam, Netherlands Organization for Health Research and Development (ZonMw), Netherlands Organisation for Scientific Research (NWO), Ministry of Health, Welfare and Sport and Ministry of Youth and Families. This project received funding from the European Union's Horizon 2020 research and innovation programme (LIFECYCLE, grant agreement No 733206, 2016; EUCAN-Connect grant agreement No 824989; ATHLETE, grant agreement No 874583). VJ received funding from a Consolidator Grant from the European Research Council (ERC-2014-CoG-648916). LD received funding from the European Union's Horizon 2020 co-funded programme ERA-Net on Biomarkers for Nutrition and Health (ERA HDHL) (ALPHABET project (no 696295; 2017), ZonMw The Netherlands (no 529051014; 2017)). JFF received received funding from the European Joint Programming Initiative "A Healthy Diet for a Healthy Life" (JPI HDHL, NutriPROGRAM project, ZonMw the Netherlands no.529051022 and PREcisE project ZonMw the Netherlands no.529051023). The study sponsors had no role in the study design, data analysis, interpretation of data, or writing of this report.

The general design, all research aims and the specific measurements in the Generation R Study have been approved by the Medical Ethical Committee of the Erasmus Medical Center, Rotterdam. New measurements will only be embedded in the study after approval of the Medical Ethical Committee. Participants are asked for their written informed consent for the four consecutive phases of the study (prenatally, birth to 4 years, 4–12 years, and from 12 years onwards). At the start of each phase, mothers and their partners receive written and oral information about the study. Even with consent of the parents, when the child is not willing to participate actively, no measurements are performed. From the age of 12 years, children are asked for written informed consent.

The authors gratefully acknowledge the contribution of participants, research collaborators, general practitioners, hospitals, midwives, and pharmacies in Rotterdam.

HGS

The Healthy Growth Study was co-funded by the European Union (European Social Fund – ESF) and Greek national funds through the Operational Program "Education and Lifelong Learning" of the National Strategic Reference Framework (NSRF) - Research Funding Program: Heracleitus II. Investing in knowledge society through the European Social Fund.

Approval to conduct the study was granted by the Greek Ministry of National Education and the Ethics Committee of Harokopio University of Athens, and the study was conducted in accordance with the ethical standards specified in the 1964 Declaration of Helsinki. Parents who agreed to the participation of their children in the study had to sign the consent form and provide their contact details.

INMA

This study was funded by grants from the Instituto de Salud Carlos III (Red INMA G03/176) and the Generalitat de Catalunya-CIRIT (1999SGR 00241). INMA-Valencia was funded by Grants from UE (FP7-ENV-2011 cod 282957 and HEALTH.2010.2.4.5-1), Spain: ISCIII (G03/176; FIS-FEDER: PI09/02647, PI11/01007, PI11/02591, PI11/02038, PI13/1944, PI13/2032, PI14/00891, PI14/01687, and PI16/1288; Miguel Servet-FEDER CP11/00178, CP15/00025, and CPII16/00051), and Generalitat Valenciana: FISABIO (UGP 15-230, UGP-15-244,and UGP-15-249). INMA-Gipuzkoa was funded by grants from the Instituto de Salud Carlos III (FISFIS PI06/0867, FISPS09/0009) 0867,Red INMA G03/176) and the Departamento de Salud del Gobierno Vasco (2005111093 and 2009111069) and the Provincial Government of Guipúzcoa (DFG06/004 and FG08/001). INM-Menorca was funded by grants from the Instituto de Salud Carlos III (Red INMA G03/176). This study was supported by funding from the European Community’s Seventh Framework Programme (FP7/2007-206) under grant agreement no 308333—the HELIX project. JJ holds Miguel Servet-II contract (CPII19/00015) awarded by the Instituto de Salud Carlos III (Co-funded by European Social Fund "Investing in your future"). ML has received funding from the European Union’s Horizon 2020 research and innovation programme under the Marie Skłodowska-Curie grant agreement No 707404. The opinions expressed in this document reflect only the author’s view. The European Commission is not responsible for any use that may be made of the information it contains. MC holds a Miguel Servet fellowship (CP16/00128) funded by Instituto de Salud Carlos III and co-funded by European Social Fund “Investing in your future". CW received a Sara Borrell fellowship (CD18/00132) from the Instituto de Salud Carlos III. RG was supported by funding from the Instituto de Salud Carlos III (PI14/00891 and PI17/00663) and Alicia Koplowitz Foundation 2017. ML has held a Miguel Servet-II contract (MSII16/00051) awarded by the Instituto de Salud Carlos III (Co-funded by European Social Fund "Investing in your future"). SL This study was supported by grants from Instituto de Salud Carlos III (FIS-FEDER: 13/1944, 16/1288 and 19/1338; Miguel Servet-FEDER: CP15/0025). MG is funded by a Miguel Servet fellowship (CP18II/00018) awarded by the Institute of Health Carlos III.

The INMA project was approved by the ethics committee in each area. All participants provided written informed consent before enrolment to the study.

The authors would particularly like to thank all the participants for their generous collaboration. The authors are grateful to Mireia Garcia, Maria Victoria Estraña, Maria Victoria Iturriaga, Cristina Capo and Josep LLuch for their assistance in contacting the families and administering the questionnaires.

MoBa

The Norwegian Mother, Father and Child Cohort Study is supported by the Norwegian Ministry of Health and Care Services and the Ministry of Education and Research.

The establishment and data collection in MoBa was previously based on a license from the Norwegian Data protection agency and approval from The Regional Committee for Medical Research Ethics, and it is now based on regulations related to the Norwegian Health Registry Act. MoBa is conducted according to the guidelines laid down in the declaration of Helsinki, and written informed consent was obtained from all participants. A detailed protocol of the study including the consent can be found elsewhere (http://www.fhi.no/morogbarn).

The current study is based on version 12 of the quality-assured data files released for research. The establishment of MoBa and initial data collection were based on a license from the Norwegian Data Protection Agency and approval from The Regional Committees for Medical and Health Research Ethics. The MoBa cohort is based on regulations of the Norwegian Health Registry Act. The current study was approved by The Regional Committees for Medical and Health Research Ethics (2018/427).

The authors are grateful to all the participating families in Norway who take part in this on-going cohort study.

NFBC1966 and NFBC1986

NFBC1966 received financial support from University of Oulu (grant numbers 65354 and 24000692), Oulu University Hospital (grant numbers 2/97, 8/97 and 24301140), Ministry of Health and Social Affairs (grant numbers 23/251/97, 160/97 and 190/97), National Institute for Health and Welfare, Helsinki (grant number 54121), Regional Institute of Occupational Health, Oulu (grant numbers 50621 and 54231) and ERDF European Regional Development Fund (grant number 539/2010 A31592). NFBC1986 received financial support from EU QLG1-CT-2000-01643 (EUROBLCS, grant number E51560), NorFA (grant numbers 731, 20056 and 30167) and USA / NIH 2000 G DF682 (grant number 50945). Financial support for data generation, research and supporting staff was received from the Academy of Finland (grants numbers: 104781, 120315, 129269, 1114194, 24300796, 285547 (EGEA)); University Hospital Oulu, Biocenter, University of Oulu, Finland (grant number: 75617); NIHM (grant number: MH063706, Smalley and Jarvelin for NFBC1986 data collection), Juselius Foundation; NFBC1966 genotyping by NHLBI (grant number: 5R01HL087679-02] through the STAMPEED program [grant number: 1RL1MH083268-01); NIH/NIMH (grant number: 5R01MH63706:02); the European Commission: EURO-BLCS, Framework 5 award QLG1-CT-2000-01643 (for NFBC1986 data collection), ENGAGE project and grant agreement HEALTH-F4-2007 (grant number: 201413); EU H2020-HCO-2004 iHEALTH Action (grant number: 643774), EU H2020-PHC-2014 DynaHealth Action (grant number: 633595); ALEC Action (grant number: 633212); ERDF European Regional Development Fund (grant number: 539/2010 A31592); the Medical Research Council (MRC), UK (grant numbers: G0500539, G0600705, G1002319, MR/M013138/1), EU H2020-SC1-2016-2017 LifeCycle Action (grant number: 733206). The programme is currently funded by EU H2020-SC1-2016-2017 LifeCycle Action (grant number: 733206) and EU-H2020 EUCAN Connect (grant number: 824989).

These studies was conducted following the principles of the Declaration of Helsinki and was approved by the Ethical Committee of Northern Ostrobothnia Hospital District. Written informed consent was obtained from all participants.

The authors thank all cohort members and researchers who have participated in the NFBC studies. We also wish acknowledge the work of the NFBC project center.

NINFEA

The NINFEA cohort was initially funded by the Compagnia SanPaolo Foundation and the Piedmont Region. It received funding from European projects: CHICOS (FP7 grant number HEALTH-FP7-2009-241604, LifeCycle (H2020 grant number 733206), ATHLETE (H2020 grant number 874583).

The Ethical Committee of the San Giovanni Battista Hospital and CTO/CRF/Maria Adelaide Hospital of Turin approved the NINFEA study (approval N. 0048362, and subsequent amendments). Informed consent was obtained from all the participants.

The authors thank all families participating in the NINFEA cohort.

RAINE Study

The Western Australian Pregnancy Cohort (Raine Study) has been funded by program and project grants from the Australian National Health and Medical Research Council, the Commonwealth Scientific and Industrial Research Organisation, Healthway, the Lions Eye Institute in Western Australia and NHMRC EU funding grant GNT114285. The University of Western Australia (UWA), Curtin University, the Raine Medical Research Foundation, the Telethon Kids Institute, the Women's and Infant's Research Foundation (KEMH), Murdoch University, The University of Notre Dame Australia and Edith Cowan University provide funding for the Core Management of the Raine Study. REF is a recipient of a National Health and Medical Research Council Early Career Fellowship.

Ethics approval was obtained from the Human Ethics Committees at King Edward Memorial Hospital, Princess Margaret Hospital, The University of Western Australia and Curtin University. All participants and guardians provided written consent.

The authors would like to acknowledge the Raine Study participants and their families. The authors would also like to acknowledge the Raine Study Team for study co-ordination and data collection, and the NH&MRC for their long term contribution to funding the study over the last 29 years.

RHEA
The "Rhea" project was financially supported by European projects (EU FP6-2003-Food-3-NewGeneris, EU FP6. STREP Hiwate, EU FP7 NV.2007.1.2.2.2. Project No 211250 Escape, EU FP7-2008-ENV-1.2.1.4 Envirogenomarkers, EU FP7-HEALTH-2009- single stage CHICOS, EU FP7 ENV.2008.1.2.1.6. Proposal No 226285 ENRIECO, EU- FP7- HEALTH-2012 Proposal No 308333 HELIX) and the Greek Ministry of Health (Program of Prevention of obesity and neurodevelopmental disorders in preschool children, in Heraklion district, Crete, Greece: 2011-2014; “Rhea Plus”: Primary Prevention Program of Environmental Risk Factors for Reproductive Health, and Child Health: 2012-15).

The study was approved by the corresponding ethical committees. All participants provided written, informed consent.

The authors would particularly like to thank all the cohort participants for their generous collaboration.

SWS
The SWS is supported by grants from the Medical Research Council, National Institute for Health, Research Southampton Biomedical Research Centre, British Heart Foundation, University of Southampton and University Hospital Southampton National Health Service Foundation Trust, and the European Union’s Seventh Framework Programme (FP7/2007-2013), project EarlyNutrition (grant 289346). Study participants were drawn from a cohort study funded by the Medical Research Council and the Dunhill Medical Trust. HMI's salary was paid by the UK Medical Research Council. Mark Hanson is supported by the British Heart Foundation.

The study had full approval from the Southampton and Southwest Hampshire Local Research Ethics Committee. All participants gave written informed consent.

The authors are grateful to the women of Southampton who gave their time to take part in the Southampton Women’s Survey and to the research nurses and other staff who collected and processed the data.

Missing data
Complete case analysis is unbiased by missing data if the chance of being a complete case is not associated with the outcome after adjusting for covariates. However, it is not possible to test this within one model, as including all covariates from a given analysis as predictors of being a complete case in that analysis would leave no variation in the variable indicating missingness. We therefore regressed a variable indicating complete cases on BMI at each age and covariates with no missingness (sex, exact age in days, cohort; Figure S2). For maternal education and pregnancy diabetes all odds ratios for the association between BMI and being a complete case were null. For area deprivation and NDVI odds ratios were close to 1 at younger ages, however at older ages higher BMI was associated with a lower chance of being a complete case.

Web Table 1: Information sources for gestational diabetes

Cohort	Source	Universal screening?
ABCD	Questionnaire, linkage with perinatal registration, and info from medical files	No
ALSPAC	Clinical records	No
BiB	OGTT	Yes
DNBC	Clinical records	No
EDEN	OGTT during study clinic and clinical record	Yes
ELFE	Clinical record	No
GECKO	Clinical records	No
Gen-R	Clinical records	No
INMA	Clinical records	No
MoBa	Questionnaire	No
NINFEA	Questionnaire	No
Raine	Questionnaire	No
Rhea	Questionnaire	No
SWS	Clinical records	No

Web Table 2: Cohort-specific methods of data collection for height and weight

Cohort	Method of height and weight measurement
ABCD	Clinical measurement
ALSPAC	Clinical measurement & parent/self-report
BiB	Clinical measurement
CHOP	Clinical measurement & parent-report
DNBC	Clinical measurement & parent-report
EDEN	Clinical measurement & parent-report
ELFE	Clinical measurement & parent-report
GECKO	Clinical measurement & parent-report
Gen-R	Clinical measurement
HGS	Clinical measurement
INMA	Clinical measurement & parent-report
MoBa	Parent and self-report
NFBC66	Medical records
NFBC86	Medical records
NINFEA	Self-report
Raine	Self-report
Rhea	Clinical measurement
SWS	Clinical measurement

Web Table 3: Numbers of observations provided by each child for each exposure

Exposure	Observations per child	N
Maternal education	1	3639
	2	4670
	3	4740
	4	3569
	5	10653
NDVI	1	1611
	2	2932
	3	3070
	4	224
	5	714
GDM	1	1372
	2	2798
	3	3354
	4	1182
	5	979

Web Table 4: Descriptive statistics for analysis dataset vs excluded participants

	**Analysis sample (N = 258568)**		**Excluded sample N = (62220)**	
	**Median (IQR) / n (%)**	**Missing, N (%)**	**Median (IQR) / n (%)**	**Missing, N (%)**
Maternal age at birth (years)	29.8 (26.6,33)	1944 (0.8)	29 (25.4,32.7)	6644 (10.7)
Area deprivation		199758 (77.3)		51592 (82.9)
Low	15055 (5.8)		2933 (4.7)	
Medium	13912 (5.4)		2589 (4.2)	
High	29843 (11.5)		5106 (8.2)	
BMI z-score age 0-1 years (KG)	-0.1 (-0.8,0.5)	38511 (14.9)	-0.1 (-0.8,0.5)	60331 (97)
BMI z-score age 2-3 years (KG)	0.4 (-0.3,1.1)	162789 (63)	0.4 (-0.2,1)	61166 (98.3)
BMI z-score age 4-7 years (KG)	0.1 (-0.5,0.8)	97235 (37.6)	0.2 (-0.4,0.9)	60741 (97.6)
BMI z-score age 8-13 years (KG)	0.1 (-0.6,0.8)	128976 (49.9)	0.3 (-0.4,1)	60813 (97.7)
BMI z-score age 14-17 years (KG)	0.1 (-0.6,0.8)	229955 (88.9)	0 (-0.6,0.7)	60962 (98)
Maternal education		17782 (6.9)		16690 (26.8)
Low	122592 (47.4)		18578 (29.9)	
Medium	73389 (28.4)		14580 (23.4)	
High	44805 (17.3)		12372 (19.9)	
Maternal ethnicity		200,673 (77.6)		55491 (89.2)
Western	41538 (65)		4460 (31.1)	
Other	16357 (25.6)		2269 (15.8)	
Gestational age (days)	280.7 (273.1,286.8)	11088 (4.3)	279.4 (271,286.2)	10870 (17.5)
Maternal height (m)	166.9 (162.8,171.2)	9725 (3.8)	166.5 (162.8,171)	12940 (20.8)
NDVI	0.4 (0.3,0.5)	198412 (76.7)	0.4 (0.3,0.5)	51335 (82.5)
Parity (Nulliparous)	138908 (53.7)	2483 (1)	30428 (48.9)	9349 (15)
Gestational diabetes (yes)	4482 (1.7)	18876 (7.3)	433 (0.7)	17369 (28)
Smoking in pregnancy (yes)	56800 (22)	6622 (2.6)	15188 (24.4)	10484 (16.8)
Maternal pre-pregnancy BMI (KG)		22107 (8.5)		15069 (24.2)
Underweight	10918 (4.2)		15188 (24.4)	
Overweight	66471 (25.7)		13601 (21.9)	
Child sex (male)	131983 (51)	0 (0)	22369 (36)	15069 (24.2)

Note: The analysis sample is defined as participants with minimum one exposure and BMI at one time point.

Web Table 5: Numbers of complete cases for each exposure-outcome combination

Exposure	BMI age period (years)	N (%) complete cases
Maternal education	0-1	206180 (64.91)
	2-3	91556 (41.46)
	4-7	151286 (47.63)
	8-13	121020 (37.78)
	14-17	27253 (19.94)
NDVI	0-2	39690 (14.85)
	3-4	22658 (13.29)
	4-7	36040 (13.48)
	8-13	29566 (11.06)
	14-17	7096 (6.31)
Gestational diabetes	0-2	177600 (60.28)
	3-4	72610 (36.71)
	4-7	124004 (42.09)
	8-13	96972 (32.91)
	14-17	12530 (10.86)

Note: denominator is the maximum n of the cohorts which contained any data for the exposure at that time poin

Web Table 6: Cohort-specific information on covariates

	Sex, n(%)		Parity, n(%)		Maternal ethnicity, n(%)			Maternal smoking in pregnancy, n(%)	
Cohort	Male	Missing	Nulliparous	Missing	Western	Other	Missing	Yes	Missing
ABCD (n = 6152)	3063 (49.8)	0 (0)	3302 (53.7)	45 (0.7)	3463 (56.3)	2644 (43)	45 (0.7)	670 (10.9)	3 (0)
ALSPAC (n=10499)	5279 (50.3)	0 (0)*	4521 (43.1)	544 (5.2)	9628 (91.7)	155 (1.5)	716 (6.8)	2332 (22.2)	1283 (12.2)
BiB (n=13400)	6920 (51.6)	0 (0)	4985 (37.2)	766 (5.7)	4685 (35)	6411 (47.8)	2304 (17.2)	1822 (13.6)	2313 (17.3)
CHOP (n=1669)	843 (50.5)	0 (0)	817 (49)	6 (0.4)	-	-	-	580 (34.8)	3 (0.2)
DNBC (n=77534)	39297 (50.7)	0 (0)	36799 (47.5)	0 (0)	-	-	-	19237 (24.8)	1068 (1.4)
EDEN (n=1765)	918 (52)	0 (0)	798 (45.2)	3 (0.2)	-	-	-	449 (25.4)	5 (0.3)
ELFE (n=17926)	9223 (51.5)	0 (0)	8120 (45.3)	250 (1.4)	12550 (70)	3141 (17.5)	2235 (12.5)	3563 (19.9)	207 (1.2)
GECKO (n=2748)	1384 (50.4)	0 (0)	1107 (40.3)	12 (0.4)	2489 (90.6)	113 (4.1)	146 (5.3)	427 (15.5)	3 (0.1)
GENR (n=8680)	4376 (50.4)	0 (0)	4660 (53.7)	251 (2.9)	4664 (53.7)	3544 (40.8)	472 (5.4)	1933 (22.3)	1231 (14.2)
HGS (n=2570)	1299 (50.5)	0 (0)	0 (0)	81 (3.2)	-	-	-	671 (26.1)	0 (0)
INMA (n=1918)	989 (51.6)	0 (0)	1030 (53.7)	62 (3.2)	1826 (95.2)	86 (4.5)	6 (0.3)	597 (31.1)	27 (1.4)
MoBa (n=85589)	43849 (51.2)	0 (0)	40826 (47.7)	71 (0.1)	-	-	-	19181 (22.4)	1 (0)
NINFEA (n=6532)	3312 (50.7)	0 (0)	4520 (69.2)	316 (4.8)	-	-	-	516 (7.9)	74 (1.1)
NFBC66 (n=7709)	4125 (53.5)	0 (0)	7 (0.1)	0 (0)	-	-	-	1602 (20.8)	122 (1.6)
NFBC86 (n=7315)	3716 (50.8)	0 (0)	2494 (34.1)	10 (0.1)	-	-	-	1779 (24.3)	8 (0.1)
Raine (n=2548)	1300 (51)	0 (0)	1191 (46.7)	52 (2)	2233 (87.6)	263 (10.3)	52 (2)	688 (27)	52 (2)
RHEA (n=1002)	526 (52.5)	0 (0)	450 (44.9)	11 (1.1)	-	-	-	302 (30.1)	84 (8.4)
SWS (n=3012)	1564 (51.9)	0 (0)	1550 (51.5)	3 (0.1)	-	-	-	451 (15)	138 (4.6)
Combined (n=258568)	131983 (51)	0 (0)	117177 (45.3)	2483 (1)	41538 (65)	16357 (25.6)	5976 (9.4)	56800 (22)	6622 (2.6)

Note: cohort ns refer to number of participants in the analysis sample (minimum one exposure and BMI at one time point)
*This may include zero.

Web Table 6: Cohort-specific information on covariates (continued)

	Maternal pre-pregnancy BMI, n(%)			Maternal age at child's birth	
Cohort	Underweight	Overweight	Missing	Median (IQR)	Missing
ABCD (n = 6152)	276 (4.5)	1274 (20.7)	485 (7.9)	32 (28, 35)	310 (5)
ALSPAC (n=10499)	993 (9.5)	1808 (17.2)	1685 (16)	29 (26, 32)	996 (9.5)
BiB (n=13400)	206 (1.5)	2333 (17.4)	8750 (65.3)	27 (23, 31)	0 (0)
CHOP (n=1669)	116 (7)	398 (23.8)	151 (9)	30 (26, 33)	9 (0.5)
DNBC (n=77534)	3132 (4)	19833 (25.6)	4817 (6.2)	30 (27, 33)	0 (0)
EDEN (n=1765)	143 (8.1)	448 (25.4)	36 (2)	29 (26, 33)	0 (0)
ELFE (n=17926)	1377 (7.7)	4799 (26.8)	296 (1.7)	30 (27, 34)	78 (0.4)
GECKO (n=2748)	50 (1.8)	956 (34.8)	191 (7)	31 (28, 34)	4 (0.1)
GENR (n=8680)	276 (3.2)	1844 (21.2)	2053 (23.7)	31 (27, 34)	0 (0)
HGS (n=2570)	154 (6)	418 (16.3)	359 (14)	28 (25, 32)	351 (13.7)
INMA (n=1918)	82 (4.3)	475 (24.8)	12 (0.6)	32 (29, 35)	8 (0.4)
MoBa (n=85589)	2487 (2.9)	25852 (30.2)	2154 (2.5)	30 (27, 33)	121 (0.1)
NINFEA (n=6532)	540 (8.3)	1218 (18.6)	143 (2.2)	33 (30, 36)	1 (0)
NFBC66 (n=7709)	201 (2.6)	1586 (20.6)	668 (8.7)	27 (22, 32)	0 (0)
NFBC86 (n=7315)	529 (7.2)	1226 (16.8)	121 (1.7)	27 (24, 31)	0 (0)
Raine (n=2548)	272 (10.7)	434 (17)	130 (5.1)	28 (24, 32)	62 (2.4)
RHEA (n=1002)	35 (3.5)	336 (33.5)	29 (2.9)	30 (26, 33)	4 (0.4)
SWS (n=3012)	49 (1.6)	1233 (40.9)	27 (0.9)	30 (27, 33)	0 (0)
Combined (n=258568)	10918 (4.2)	66471 (25.7)	22107 (8.5)	29.8 (26.6, 33)	1944 (0.8)

Note: cohort ns refer to number of participants in the analysis sample (minimum one exposure and BMI at one time point)

Web Table 7: Child BMI z-scores by cohort

	0-1 years		2-3 years		4-7 years		8-13 years		14-17 years	
**Cohort**	**n**	**BMI z-score, median (IQR)**	**n**	**BMI z-score, median (IQR)**	**n**	**BMI z-score, median (IQR)**	**n**	**BMI z-score, median (IQR)**	**n**	**BMI z-score, median (IQR)**
ABCD (n = 6152)	5669	-0.1 (-0.7, 0.6)	4763	0.3 (-0.3, 1)	4754	0.2 (-0.5, 0.8)	3603	0 (-0.7, 0.9)	-	-
ALSPAC (n=10499)	1420	0.1 (-0.6, 0.7)	1221	0.7 (0, 1.3)	5682	0.3 (-0.4, 1)	9585	0.3 (-0.4, 1.1)	7675	0.1 (-0.5, 0.9)
BiB (n=13400)	12959	-0.6 (-1.3, 0.1)	6225	0.5 (-0.2, 1.2)	10539	0.4 (-0.3, 1.1)	5592	0.2 (-0.7, 1.3)	-	-
CHOP (n=1669)	1668	-0.5 (-1.1, 0.1)	938	0.1 (-0.5, 0.8)	1092	0.3 (-0.3, 0.9)	755	0.3 (-0.4, 1.2)	-	-
DNBC (n=77534)	56821	-0.3 (-1, 0.4)	-	-	43164	0 (-0.6, 0.6)	44177	-0.2 (-0.9, 0.6)	6508	0.2 (-0.4, 0.9)
EDEN (n=1765)	1760	-1.4 (-2.6, -0.2)	1521	0.1 (-0.6, 0.7)	1278	0 (-0.5, 0.7)	904	-0.1 (-0.8, 0.7)	-	-
ELFE (n=17926)	17795	0 (-0.7, 0.7)	10773	0 (-0.7, 0.7)	10192	0 (-0.6, 0.6)	3360	-0.1 (-0.8, 0.6)	-	-
GECKO (n=2748)	2738	0 (-0.6, 0.6)	2212	0.4 (-0.3, 0.9)	2309	0.4 (-0.2, 0.9)	2180	0.2 (-0.5, 1)	-	-
GENR (n=8680)	7230	0 (-0.7, 0.7)	6466	0.5 (-0.2, 1.1)	6572	0.3 (-0.2, 1)	5723	0.3 (-0.4, 1.1)	-	-
HGS (n=2570)	-	-	-	-	-	-	2568	1 (0.1, 1.8)	-	-
INMA (n=1918)	1910	-0.2 (-0.9, 0.3)	1177	0.4 (-0.3, 1.1)	1634	0.5 (-0.1, 1.2)	1043	0.8 (-0.1, 1.7)	-	-
MoBa (n=85589)	85079	0 (-0.7, 0.7)	45673	0.4 (-0.3, 1.1)	49728	0.1 (-0.5, 0.8)	33473	0.1 (-0.6, 0.9)	-	-
NINFEA (n=6532)	6269	-0.4 (-1.3, 0.4)	255	0.3 (-0.6, 0.9)	4870	0.1 (-0.7, 0.9)	1109	0.1 (-0.7, 0.9)	-	-
NFBC66 (n=7709)	7379	-0.2 (-0.9, 0.6)	5809	0.6 (-0.1, 1.2)	7268	0.1 (-0.5, 0.7)	7239	0 (-0.6, 0.6)	7035	-0.1 (-0.8, 0.5)
NFBC86 (n=7315)	5141	-0.1 (-0.8, 0.4)	4739	0.5 (-0.1, 1.1)	7110	0.3 (-0.3, 0.9)	4750	0.2 (-0.4, 1)	5760	0 (-0.6, 0.7)
Raine (n=2548)	2303	0.4 (-0.2, 1.1)	614	0 (-0.6, 0.7)	2088	0.2 (-0.4, 0.8)	1988	0.3 (-0.3, 1.2)	1623	0.4 (-0.3, 1.2)
RHEA (n=1002)	974	-0.6 (-1.3, 0.1)	684	0 (-0.7, 0.9)	887	0.6 (-0.1, 1.3)	334	1.1 (0.2, 1.9)	-	-
SWS (n=3012)	2942	0.4 (-0.3, 1)	2701	0.7 (0, 1.3)	2166	0.3 (-0.2, 1)	1209	0.1 (-0.7, 1)	-	-
Combined (n=258568)	220057	-0.1 (-0.8, 0.5)	95779	0.4 (-0.3, 1.1)	161333	0.1 (-0.5, 0.8)	129592	0.1 (-0.6, 0.8)	28613	0.1 (-0.6, 0.8)

Note: cohort ns refer to number of participants in the analysis sample (minimum one exposure and BMI at one time point)

Web Table 8: Child height measurements (cm) by cohort

	0-1 years		2-3 years		4-7 years		8-13 years		14-17 years	
**Cohort**	**n**	**Height, median (IQR)**	**n**	**Height, median (IQR)**	**n**	**Height, median (IQR)**	**n**	**Height, median (IQR)**	**n**	**Height, median (IQR)**
ABCD (n = 6152)	5669	55.5 (53.9, 57.6)	4763	91.5 (88.5, 95.5)	4754	112 (107, 117.4)	3603	146.5 (141.2, 151.8)	-	-
ALSPAC (n=10499)	1420	63.5 (61.7, 67.8)	1221	87.5 (85.2, 90)	5682	114 (109, 119)	9585	132.4 (128, 137.9)	7675	168 (162, 173.3)
BiB (n=13400)	12959	53 (51, 56)	6225	89 (86, 94)	10539	106.9 (103.4, 110.6)	5592	130.5 (126.3, 135)	-	-
CHOP (n=1669)	1668	51.6 (50, 54)	938	89 (86.6, 91.6)	1092	107 (103, 115)	755	137.2 (128.9, 147.8)	-	-
DNBC (n=77534)	56821	68 (66.5, 70)	-	-	43164	125.5 (122, 129)	44177	151 (145, 156)	6508	172 (167, 179)
EDEN (n=1765)	1760	54 (52, 56)	1521	89.5 (86.5, 93)	1278	107.5 (104, 112)	904	134 (128, 142.6)	-	-
ELFE (n=17926)	17795	50 (48, 51)	10773	90 (87, 96)	10192	108 (104, 113)	3360	131 (127, 135)	-	-
GECKO (n=2748)	2738	55 (53.5, 57)	2212	91 (88, 95)	2309	117.5 (112.5, 121.5)	2180	148 (143, 152.5)	-	-
GENR (n=8680)	7230	56 (53.5, 61)	6466	90 (87, 93.5)	6572	118.5 (114.8, 122.7)	5723	141.1 (136.8, 145.7)	-	-
HGS (n=2570)	-	-	-	-	-	-	2568	148.5 (143.2, 153.9)	-	-
INMA (n=1918)	1910	51.5 (50, 53)	1177	89.5 (86.5, 93.5)	1634	104.5 (101.5, 107.5)	1043	135.1 (130.5, 140.1)	-	-
MoBa (n=85589)	85079	58 (56, 61)	45673	92 (88, 96)	49728	116 (111, 124)	33473	132 (128, 136)	-	-
NINFEA (n=6532)	6269	61 (59, 65)	255	91 (88, 98)	4870	104 (100, 106)	1109	140 (135, 147)	-	-
NFBC66 (n=7709)	7379	60 (56, 65)	5809	89 (86, 93)	7268	110 (104, 117)	7239	133 (127.5, 140)	7035	163 (158, 168.5)
NFBC86 (n=7315)	5141	56.6 (54.5, 59)	4739	89 (86.5, 93)	7110	109.5 (104, 120)	4750	133.5 (129, 138.1)	5760	166 (160.8, 172)
Raine (n=2548)	2303	77.5 (75.6, 79.5)	614	90 (87.6, 92.2)	2088	116.2 (112.8, 119.5)	1988	134.5 (129, 141.8)	1623	165 (160, 172)
RHEA (n=1002)	974	53 (51, 55)	684	92 (89, 95)	887	105.1 (102, 108)	334	144.6 (140.2, 150.5)	-	-
SWS (n=3012)	2942	68.4 (66.3, 71.5)	2701	88.3 (85.4, 92.6)	2166	109.7 (103.5, 120.4)	1209	135.1 (131, 139.3)	-	-
Combined (n=258568)	220057	59.8 (57.9, 62.5)	95779	90.8 (87.4, 95)	161333	116.1 (111.7, 121.8)	129592	140.2 (135.2, 145.1)	28613	167.1 (161.8, 173.1)

Note: cohort ns refer to number of participants in the analysis sample (minimum one exposure and BMI at one time point)

Web Table 9: Child weight measurements (kg) by cohort

	0-1 years		2-3 years		4-7 years		8-13 years		14-17 years	
**Cohort**	**n**	**Weight, median (IQR)**	**n**	**Weight, median (IQR)**	**n**	**Weight, median (IQR)**	**n**	**Weight, median (IQR)**	**n**	**Weight, median (IQR)**
ABCD (n = 6152)	5669	4.7 (4.2, 5.2)	4763	13.6 (12.5, 15)	4754	19.5 (17.5, 21.7)	3603	36.6 (32.5, 42)	-	-
ALSPAC (n=10499)	1420	7 (6.3, 8)	1221	12.8 (11.9, 13.9)	5682	20 (18.1, 22)	9585	29 (26, 34)	7675	58 (51.3, 65.3)
BiB (n=13400)	12959	3.6 (3.2, 4.3)	6225	13.2 (12, 14.8)	10539	18.1 (16.4, 20)	5592	27.7 (24.3, 32.9)	-	-
CHOP (n=1669)	1668	3.5 (3.1, 4)	938	12.7 (11.7, 13.7)	1092	18.2 (16.4, 21)	755	32.4 (27, 40.6)	-	-
DNBC (n=77534)	56821	7.8 (7.1, 8.5)	-	-	43164	24.2 (22, 27)	44177	39 (34, 45)	6508	65 (58, 75)
EDEN (n=1765)	1760	3.6 (3.1, 4.4)	1521	12.8 (11.8, 14)	1278	18 (16.2, 19.8)	904	29 (25, 35)	-	-
ELFE (n=17926)	17795	3.4 (3, 3.7)	10773	13 (11.8, 14.5)	10192	18 (16.3, 20)	3360	27 (24.6, 30.4)	-	-
GECKO (n=2748)	2738	4.6 (4.2, 5.2)	2212	13.5 (12.5, 14.9)	2309	21.6 (19.5, 23.9)	2180	37.6 (33.7, 43.2)	-	-
GENR (n=8680)	7230	4.8 (4.2, 6.1)	6466	13.3 (12.2, 14.6)	6572	22.2 (20.2, 24.8)	5723	33.8 (30.2, 38.8)	-	-
HGS (n=2570)	-	-	-	-	-	-	2568	43.5 (37.1, 52)	-	-
INMA (n=1918)	1910	3.6 (3.2, 4)	1177	13.1 (11.9, 14.5)	1634	17.4 (16, 19.2)	1043	32.4 (27.6, 38.8)	-	-
MoBa (n=85589)	85079	5.4 (4.8, 6.1)	45673	13.8 (12.5, 15)	49728	21 (19, 24)	33473	28 (25, 31)	-	-
NINFEA (n=6532)	6269	6 (5.4, 7)	255	13.5 (12, 15)	4870	16 (15, 18)	1109	34 (29.3, 40)	-	-
NFBC66 (n=7709)	7379	5.7 (4.6, 7.3)	5809	13.1 (12, 14.5)	7268	18.5 (16.6, 21.2)	7239	29 (25, 33.5)	7035	52 (46.3, 58)
NFBC86 (n=7315)	5141	4.9 (4.3, 5.6)	4739	13.1 (12, 14.4)	7110	19 (16.8, 23)	4750	29.6 (26.1, 34)	5760	56 (50, 63.2)
Raine (n=2548)	2303	10.2 (9.4, 11.1)	614	12.8 (11.8, 14)	2088	21 (19.1, 23.2)	1988	30.8 (26.5, 36.9)	1623	57.9 (50.9, 67.5)
RHEA (n=1002)	974	3.7 (3.3, 4.2)	684	13.3 (12.2, 14.6)	887	17.8 (16.2, 19.8)	334	41.5 (35.3, 49.4)	-	-
SWS (n=3012)	2942	8.3 (7.5, 9.3)	2701	13.1 (12, 14.4)	2166	19.5 (16.8, 22.9)	1209	29.7 (26.7, 34.3)	-	-
Combined (n=258568)	214388	5.8 (5.2, 6.5)	91016	13.5 (12.2, 14.8)	156579	21 (19, 23.7)	125989	33 (29, 37.9)	28613	57.7 (51.3, 65.4)

Note: cohort ns refer to number of participants in the analysis sample (minimum one exposure and BMI at one time point)

Web Table 10: Age at height and weight measurement (months) by cohort

	0-1 years		2-3 years		4-7 years		8-13 years		14-17 years	
**Cohort**	**n**	**Child age, median (IQR)**	**n**	**Child age, median (IQR)**	**n**	**Child age, median (IQR)**	**n**	**Child age, median (IQR)**	**n**	**Child age, median (IQR)**
ABCD (n = 6152)	5669	1.1 (1.1, 1.5)	4763	28 (25.8, 33)	4754	63.2 (50.9, 68.4)	3603	127.9 (123.9, 132.5)	-	-
ALSPAC (n=10499)	1420	3.9 (3.7, 8)	1221	24.8 (24.8, 25.1)	5682	69 (69, 70)	9585	102 (98, 109)	7675	177 (175, 186)
BiB (n=13400)	12959	0.3 (0.2, 1.4)	6225	25.7 (24.6, 36)	10539	55.8 (51.8, 59.6)	5592	100.7 (98.3, 103.2)	-	-
CHOP (n=1669)	1668	0.5 (0.1, 0.9)	938	24.2 (24.1, 29.5)	1092	53.9 (48.4, 72.1)	755	99 (96.7, 133.9)	-	-
DNBC (n=77534)	56821	5.2 (5, 5.5)	-	-	43164	84 (84, 85.2)	44177	133.2 (132, 135.6)	6508	210.3 (210, 210.7)
EDEN (n=1765)	1760	1 (0.9, 1.1)	1521	24.8 (24.2, 29.8)	1278	55.1 (49.5, 64.7)	904	100.3 (96.8, 125.8)	-	-
ELFE (n=17926)	17795	0 (0, 0)	10773	25.2 (24.2, 37.4)	10192	56.1 (50.9, 63.3)	3360	103.1 (101.1, 106.1)	-	-
GECKO (n=2748)	2738	1.2 (1, 1.5)	2212	25.9 (24.9, 28.6)	2309	69.5 (65.3, 72.6)	2180	127.3 (123.7, 131)	-	-
GENR (n=8680)	7230	1.2 (1, 3.3)	6466	25.8 (24.7, 30.2)	6572	72.1 (70.2, 74.9)	5723	116.7 (115.3, 118.3)	-	-
HGS (n=2570)	-	-	-	-	-	-	2568	133.9 (127.2, 139.6)	-	-
INMA (n=1918)	1910	0.4 (0.3, 0.6)	1177	24.9 (24.1, 32.9)	1634	50.4 (48.8, 52.6)	1043	109.1 (105.2, 113.3)	-	-
MoBa (n=85589)	85079	1.6 (1.4, 3)	45673	27.7 (25, 36.2)	49728	63 (61, 84)	33473	97 (97, 98)	-	-
NINFEA (n=6532)	6269	3 (3, 3)	255	28.6 (25.4, 46.7)	4870	48 (48, 48.7)	1109	121.9 (120.6, 124.9)	-	-
NFBC66 (n=7709)	7379	2.4 (1.2, 4.8)	5809	26.4 (24, 33.6)	7268	60 (52.8, 78)	7239	106.8 (100.8, 127.2)	7035	174 (170.4, 177.6)
NFBC86 (n=7315)	5141	1.5 (1.1, 2)	4739	24.9 (24.3, 35.4)	7110	60 (48.9, 83)	4750	106.4 (100.8, 113.2)	5760	180.2 (174.1, 189)
Raine (n=2548)	2303	13.7 (12.9, 14.5)	614	25.8 (25.2, 26.8)	2088	71 (70.1, 72.4)	1988	102.2 (98.5, 125.5)	1623	170 (169.1, 172.1)
RHEA (n=1002)	974	0.6 (0.4, 1)	684	25.9 (24.6, 29.2)	887	49.7 (49.1, 50.7)	334	131.4 (130.1, 134.2)	-	-
SWS (n=3012)	2942	6.3 (6, 8)	2701	24.8 (24.3, 35.7)	2166	50.6 (49.2, 81)	1209	109.8 (107.8, 111.9)	-	-
Combined (n=258568)	220057	2.5 (2.3, 3.5)	95779	26.7 (24.7, 34.9)	161333	67.3 (64.6, 77.6)	129592	114.5 (112.6, 118.9)	28613	184.1 (181.3, 189.4)

Note: cohort ns refer to number of participants in the analysis sample (minimum one exposure and BMI at one time point)

Web Table 11: GDM analysis stratified by test type

	Questionnaire or non-universal test				Universal blood-based test			
Age	N cohorts	N unexposed	N exposed	Estimate	N cohorts	N unexposed	N exposed	Estimate
0-1	12	169073	2754	0.07 (-0.02, 0.17)	2	5328	445	-0.19 (-0.42, 0.04)
2-3	11	67856	1160	0.04 (-0.02, 0.1)	2	3317	277	-0.04 (-0.17, 0.09)
4-7	12	117624	1836	0.06 (-0.03, 0.14)	2	4183	361	0.03 (-0.17, 0.23)
8-13	12	93535	966	0.19 (0.11, 0.27)	2	2275	196	0.10 (-0.09, 0.28)

*Note: no cohorts with data available at 14-17 assessed GDM via universal blood-based test. Models adjusted for child sex, exact age at measurement, maternal education, parity and pre-pregnancy BMI.

Web Table 12: Analysis on subgroup with ethnicity data

Exposure	Age	N	n	Original model	Additionally adjusting for ethnicity
Maternal education (ref = high)					
Medium	0-1	8	45601	0.02 (0,0.04)	0.03 (0.01,0.05)
	2-3	8	30752	0.09 (0.06,0.12)	0.08 (0.06,0.11)
	4-7	8	39718	0.15 (0.12,0.17)	0.15 (0.12,0.17)
	8-13	8	30214	0.32 (0.29,0.35)	0.32 (0.29,0.35)
	14-17	2	8717	0.06 (0,0.12)	0.06 (0,0.12)
Low	0-1	8	45601	-0.19 (-0.22,-0.17)	-0.15 (-0.17,-0.12)
	2-3	8	30752	0.24 (0.21,0.27)	0.21 (0.17,0.24)
	4-7	8	39718	0.31 (0.28,0.33)	0.29 (0.26,0.31)
	8-13	8	30214	0.38 (0.34,0.41)	0.36 (0.32,0.39)
	14-17	2	8717	0.29 (0.21,0.36)	0.29 (0.21,0.36)
NDVI	0-1	5	23248	-0.01 (-0.02,0.01)	-0.01 (-0.03,0)
	2-3	5	16267	0.05 (0.03,0.07)	0.05 (0.03,0.07)
	4-7	5	23403	0.03 (0.01,0.05)	0.03 (0.01,0.05)
	8-13	5	20466	-0.02 (-0.05,0)	-0.02 (-0.05,0)
	14-17	1	6189	0.03 (-0.01,0.08)	0.03 (-0.01,0.08)
GDM	0-1	8	35661	-0.04 (-0.1,0.01)	-0.03 (-0.09,0.02)
	2-3	8	24635	-0.09 (-0.16,-0.03)	-0.1 (-0.17,-0.04)
	4-7	8	30755	-0.08 (-0.14,-0.02)	-0.09 (-0.16,-0.03)
	8-13	8	23205	0.06 (-0.04,0.16)	0.05 (-0.05,0.14)
	14-17	2	6965	0.18 (-0.11,0.47)	0.18 (-0.11,0.47)

Web Table 13: Analyses with DNBC & MoBa removed

		Full model			Excluding DNBC & MoBa		
Exposure	Age	N	n	Estimate	N	n	Estimate
Maternal education (ref = high)							
Medium	0-1	17	206180	0.01 (0, 0.02)	15	73949	0.03 (0.01, 0.05)
	2-3	16	91556	-0.02 (-0.04, -0.01)	15	47945	0.01 (-0.01, 0.04)
	4-7	17	151286	0.09 (0.08, 0.1)	15	64934	0.10 (0.08, 0.12)
	8-13	18	121020	0.15 (0.13, 0.16)	16	49297	0.19 (0.17, 0.22)
Low	0-1	17	206180	0.02 (0, 0.03)	15	73949	0.04 (0.02, 0.06)
	2-3	16	91556	0.03 (0, 0.05)	15	47945	0.06 (0.03, 0.08)
	4-7	17	151286	0.16 (0.14, 0.17)	15	64934	0.14 (0.11, 0.16)
	8-13	18	121020	0.24 (0.22, 0.26)	16	49297	0.25 (0.22, 0.28)
NDVI	0-1	10	39690	0.05 (0.03, 0.06)	8	27330	0.05 (0.03, 0.07)
	2-3	9	22658	0.02 (0, 0.04)	8	18118	0.03 (0.01, 0.05)
	4-7	10	36040	0.04 (0.02, 0.05)	8	26632	0.05 (0.03, 0.07)
	8-13	10	29566	0.04 (0.01, 0.06)	8	21982	0.04 (0.02, 0.07)
GDM	0-1	14	177600	0 (-0.04, 0.04)	12	48452	0 (-0.04, 0.05)
	2-3	13	72610	0.03 (-0.03, 0.08)	12	29923	0.02 (-0.04, 0.08)
	4-7	14	124004	0.01 (-0.03, 0.06)	12	39506	-0.02 (-0.08, 0.03)
	8-13	14	96972	0.18 (0.12, 0.25)	12	26700	0.15 (0.06, 0.24)

Note: N = number of studies, n = number of participants. Ages 14-17 not shown because MoBa did not contribute to these analysis

Web Figure 1: Directed acyclic graphs

Figure S1a: Maternal education

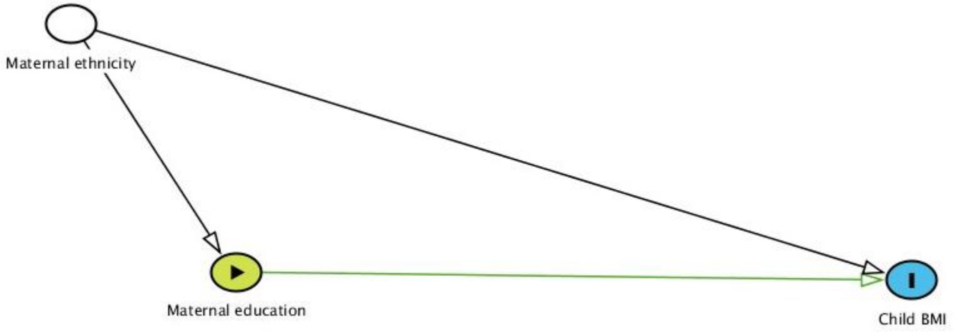

Figure S1b: Green spaces (NDVI)

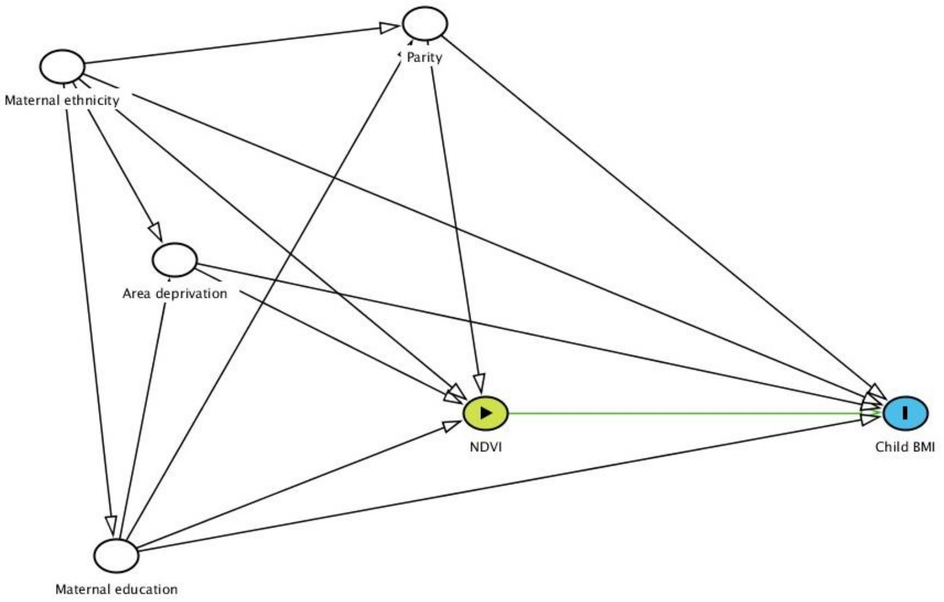

Figure S1c: Gestational Diabetes

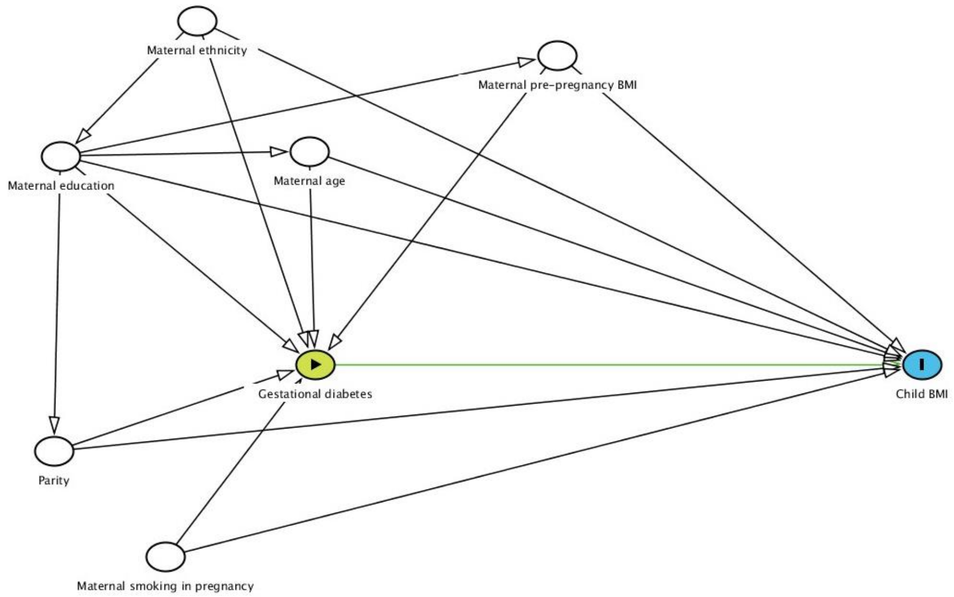

eing a compete case

Figure S2b: NDVI

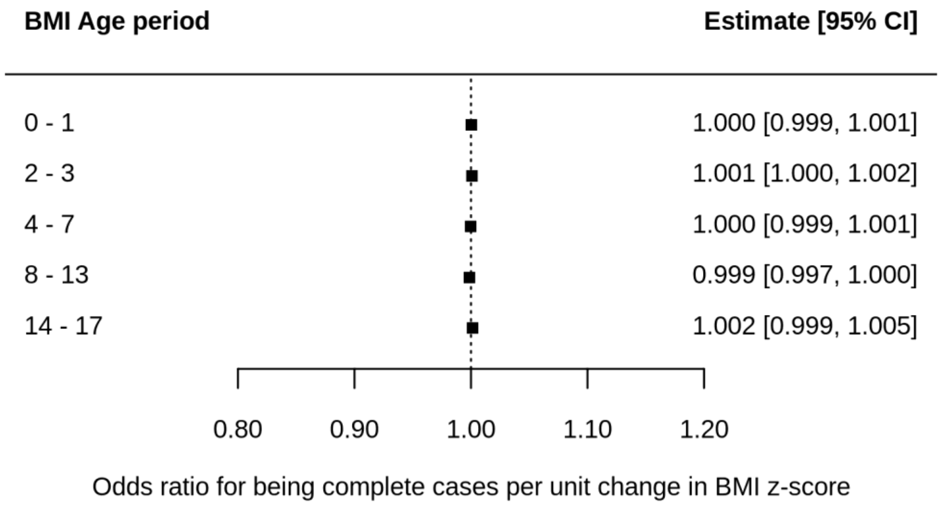

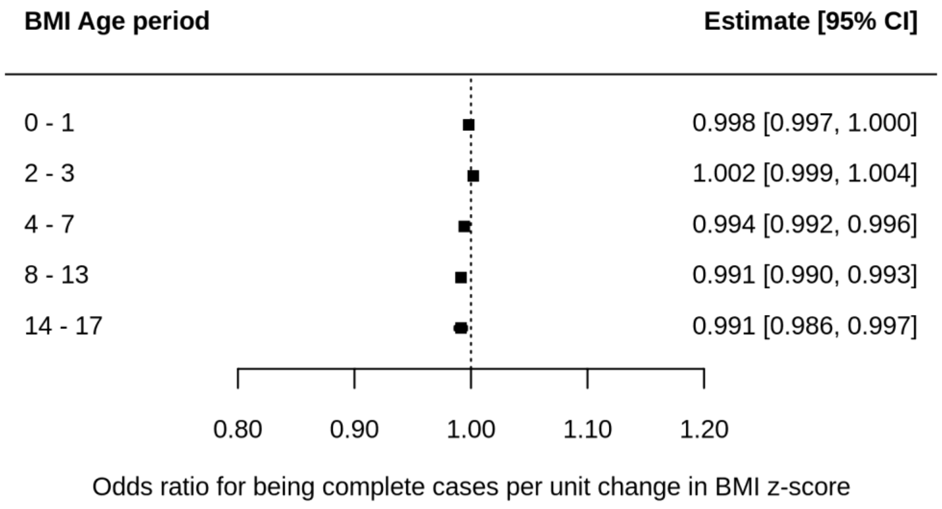

Figure S2c: Gestational diabetes

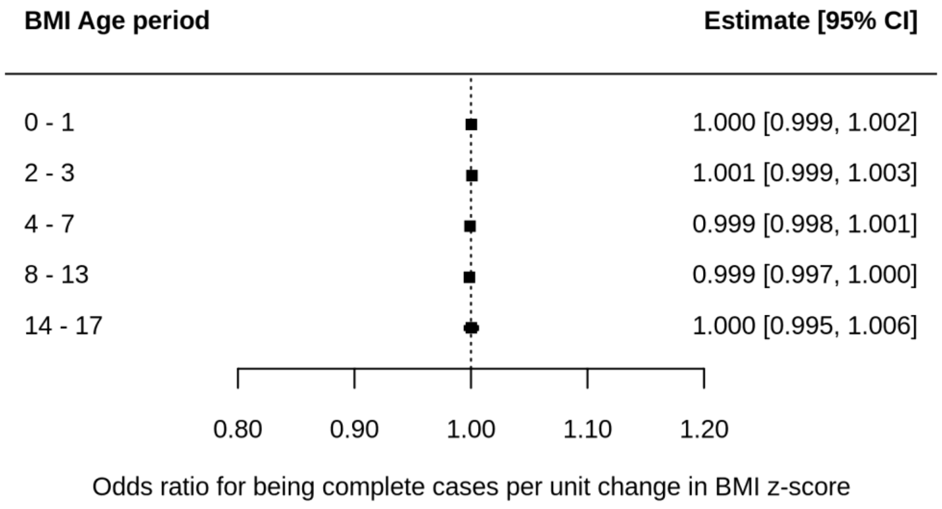

Web Figure 3a: Associations between maternal education and child BMI z-scores using 2-stage IPD meta-analysis (medium education vs high)

Cohort	N participants High education	N participants Med education	Study weight	Estimate [95% CI]
**0-1 years**				
ABCD	2931	1490	3.28	-0.04 [-0.10, 0.02]
ALSPAC	200	964	0.48	0.14 [-0.02, 0.29]
BiB	2725	1566	2.77	0.04 [-0.02, 0.11]
CHOP	403	835	1.13	0.04 [-0.06, 0.14]
DNBC	26231	11455	19.09	-0.00 [-0.03, 0.02]
EDEN	967	677	0.56	0.08 [-0.07, 0.22]
ELFE	10127	6169	9.3	0.05 [0.01, 0.08]
GECKO	928	744	1.45	0.05 [-0.04, 0.14]
GENR	3195	2783	3.51	-0.06 [-0.11, 0.00]
INMA	664	784	1.22	-0.02 [-0.11, 0.08]
MoBa	53431	25694	49.15	0.01 [-0.01, 0.02]
NFBC66	254	1022	0.59	0.05 [-0.09, 0.19]
NFBC86	1270	1919	2.31	0.01 [-0.06, 0.08]
NINFEA	3911	2021	2.2	0.06 [-0.01, 0.13]
Raine	428	592	0.75	0.06 [-0.07, 0.18]
RHEA	308	491	0.44	0.14 [-0.03, 0.30]
SWS	844	1738	1.79	0.16 [0.08, 0.24]
RE Model (I^2 = 65.5)				0.03 [0.00, 0.05]
**2-3 years**				
ABCD	2485	1259	5.47	-0.01 [-0.08, 0.06]
ALSPAC	169	859	1.05	0.06 [-0.10, 0.21]
BiB	1374	808	2.65	0.09 [-0.01, 0.19]
CHOP	257	479	1.18	-0.07 [-0.21, 0.08]
EDEN	881	558	2.46	0.09 [-0.02, 0.19]
ELFE	7233	3041	12.46	0.02 [-0.02, 0.07]
GECKO	763	625	2.66	0.06 [-0.04, 0.16]
GENR	2898	2460	9.11	-0.03 [-0.09, 0.02]
INMA	462	467	1.46	-0.16 [-0.30, -0.03]
MoBa	30550	12413	49.79	-0.06 [-0.08, -0.03]
NFBC66	220	857	1.28	0.05 [-0.09, 0.19]
NFBC86	1160	1780	5.48	-0.06 [-0.13, 0.00]
NINFEA	165	80	0.23	0.04 [-0.30, 0.38]
Raine	129	174	0.51	0.04 [-0.19, 0.26]
RHEA	222	348	0.58	0.03 [-0.18, 0.24]
SWS	778	1602	3.63	0.02 [-0.06, 0.11]
RE Model (I^2 = 51.8)				-0.00 [-0.03, 0.03]
**4-7 years**				
ABCD	2477	1272	4.11	0.19 [0.12, 0.25]
ALSPAC	931	3925	2.17	-0.02 [-0.11, 0.07]
BiB	2142	1228	2.92	0.06 [-0.02, 0.14]
CHOP	304	553	0.76	-0.01 [-0.17, 0.14]
DNBC	21004	8374	21.32	0.08 [0.05, 0.11]
EDEN	763	455	1.52	0.11 [-0.00, 0.22]
ELFE	6976	2801	8.83	0.07 [0.02, 0.12]
GECKO	784	645	2.07	0.04 [-0.05, 0.14]
GENR	2833	2615	6.75	0.23 [0.17, 0.28]
INMA	591	676	1.3	0.04 [-0.08, 0.16]
MoBa	33922	12882	35.48	0.08 [0.06, 0.10]
NFBC66	249	1009	1.13	0.04 [-0.09, 0.16]
NFBC86	1685	2656	4.93	0.01 [-0.05, 0.08]
NINFEA	3111	1538	3.05	0.06 [-0.02, 0.14]
Raine	415	548	0.97	-0.07 [-0.20, 0.07]
RHEA	286	453	0.54	0.11 [-0.07, 0.29]
SWS	654	1289	2.15	0.15 [0.06, 0.24]
RE Model (I^2 = 77.7)				0.08 [0.04, 0.11]
**8-13 years**				
ABCD	1961	916	3.36	0.46 [0.37, 0.55]
ALSPAC	1348	6301	3.92	0.15 [0.06, 0.23]
BiB	1065	629	1.32	0.11 [-0.02, 0.25]
CHOP	221	383	0.63	0.14 [-0.06, 0.34]
DNBC	22362	8287	34.16	0.09 [0.07, 0.12]
EDEN	586	288	1.11	0.25 [0.10, 0.41]
ELFE	2706	600	2.69	0.08 [-0.02, 0.18]
GECKO	769	628	1.94	0.11 [-0.01, 0.22]
GENR	2658	2231	6.93	0.39 [0.33, 0.45]
HGS	1204	822	2.2	0.03 [-0.07, 0.14]
INMA	344	443	0.81	0.33 [0.15, 0.50]
MoBa	23242	8338	31.97	0.13 [0.10, 0.16]
NFBC66	249	986	1.35	0.08 [-0.06, 0.22]
NFBC86	1159	1772	3.94	0.03 [-0.05, 0.11]
NINFEA	687	371	1.03	0.12 [-0.03, 0.28]
Raine	386	531	1.1	-0.06 [-0.22, 0.09]
RHEA	129	167	0.31	0.08 [-0.21, 0.36]
SWS	387	704	1.21	0.22 [0.07, 0.36]
RE Model (I^2 = 90.8)				0.16 [0.09, 0.22]
**14-17 years**				
ALSPAC	1201	5102	30.74	0.12 [0.05, 0.18]
DNBC	2927	1245	27.2	0.12 [0.05, 0.19]
NFBC66	250	978	7.81	0.09 [-0.05, 0.22]
NFBC86	1434	2160	29.26	0.05 [-0.02, 0.12]
Raine	315	453	4.98	-0.09 [-0.26, 0.08]
RE Model (I^2 = 0.3)				0.09 [0.05, 0.12]

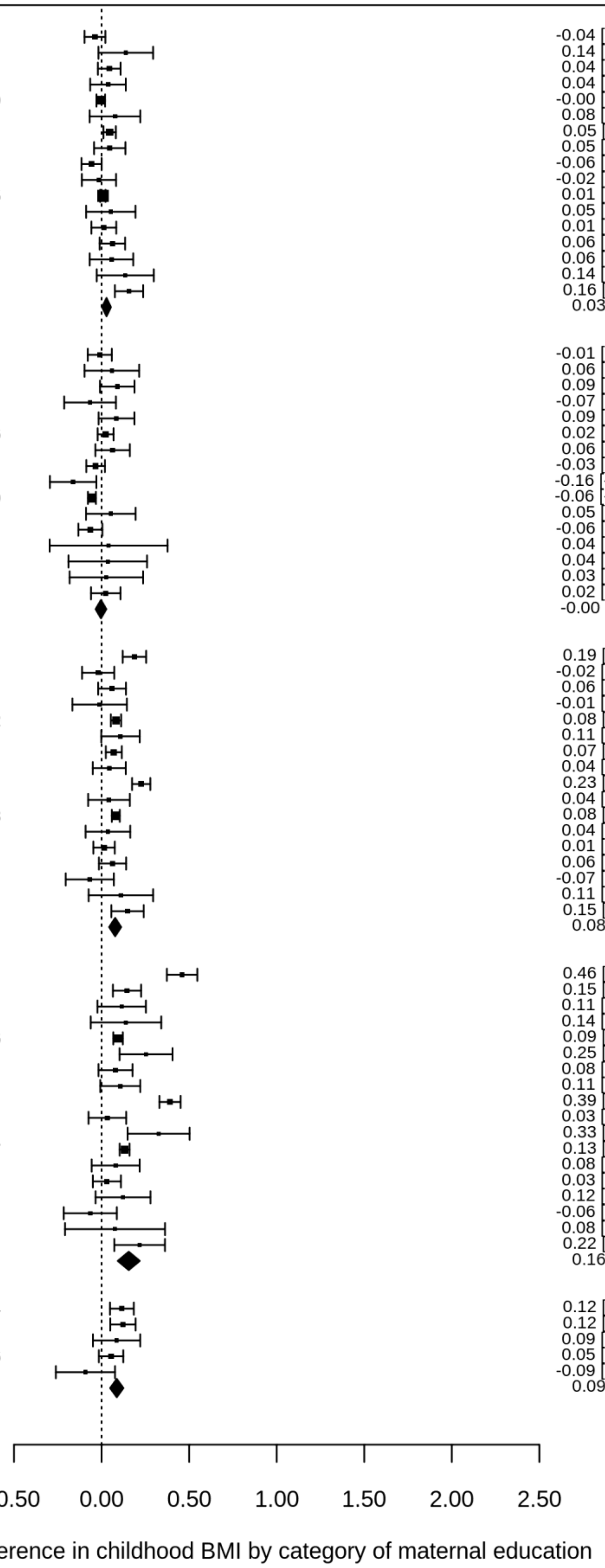

Note: models adjusted for child sex and exact age at measurement.

Web Figure 3b: Associations between maternal education and child BMI z-scores using 2-stage IPD meta-analysis (low education vs high)

Cohort	N participants: High education	N participants: Low education	Study weight	Estimate [95% CI]
**0-1 years**				
ABCD	2931	1199	5.89	0.06 [-0.01, 0.12]
ALSPAC	200	200	0.6	0.04 [-0.16, 0.24]
BiB	2725	5611	10.64	0.00 [-0.05, 0.05]
CHOP	403	423	1.78	-0.03 [-0.15, 0.09]
DNBC	26231	13631	44.84	0.01 [-0.02, 0.03]
EDEN	967	106	0.28	0.28 [-0.01, 0.58]
ELFE	10127	1488	6.57	0.06 [-0.00, 0.12]
GECKO	928	924	3.4	0.04 [-0.04, 0.13]
GENR	3195	596	2.47	0.19 [0.09, 0.28]
INMA	664	458	1.91	-0.03 [-0.14, 0.09]
MoBa	53431	1789	10.24	0.00 [-0.04, 0.05]
NFBC66	254	6103	1.46	0.14 [0.01, 0.27]
NFBC86	1270	1952	4.86	-0.04 [-0.11, 0.03]
NINFEA	3911	293	0.94	-0.12 [-0.28, 0.04]
Raine	428	1124	1.96	-0.02 [-0.13, 0.09]
RHEA	308	170	0.54	0.09 [-0.12, 0.30]
SWS	844	352	1.63	0.11 [-0.02, 0.23]
RE Model (I^2 = 46.1)				0.03 [0.00, 0.05]
**2-3 years**				
ABCD	2485	983	13.57	0.15 [0.08, 0.23]
ALSPAC	169	157	1.79	-0.03 [-0.24, 0.18]
BiB	1374	2717	14.01	-0.00 [-0.07, 0.07]
CHOP	257	200	2.3	-0.05 [-0.23, 0.13]
EDEN	881	74	1.45	0.01 [-0.22, 0.24]
ELFE	7233	499	7.99	0.20 [0.10, 0.30]
GECKO	763	722	8.46	0.06 [-0.03, 0.16]
GENR	2898	511	8.76	0.19 [0.10, 0.29]
INMA	462	245	2.94	-0.12 [-0.29, 0.04]
MoBa	30550	648	10.56	-0.09 [-0.18, -0.01]
NFBC66	220	4732	4.54	0.07 [-0.06, 0.19]
NFBC86	1160	1799	16.23	-0.04 [-0.11, 0.03]
NINFEA	165	9	0.11	0.16 [-0.69, 1.01]
Raine	129	280	1.8	-0.02 [-0.22, 0.19]
RHEA	222	111	0.95	0.18 [-0.10, 0.46]
SWS	778	313	4.56	0.09 [-0.04, 0.22]
RE Model (I^2 = 68.4)				0.04 [-0.01, 0.10]
**4-7 years**				
ABCD	2477	972	6.42	0.40 [0.32, 0.47]
ALSPAC	931	713	2.2	0.04 [-0.09, 0.16]
BiB	2142	4700	10.4	0.07 [0.02, 0.13]
CHOP	304	232	0.96	0.15 [-0.04, 0.34]
DNBC	21004	9536	44.1	0.17 [0.15, 0.20]
EDEN	763	55	0.52	-0.04 [-0.29, 0.22]
ELFE	6976	415	3.27	0.13 [0.02, 0.23]
GECKO	784	758	4.24	0.20 [0.11, 0.29]
GENR	2833	552	4.31	0.55 [0.46, 0.64]
INMA	591	363	1.75	-0.01 [-0.15, 0.13]
MoBa	33922	634	4.49	0.20 [0.11, 0.29]
NFBC66	249	6010	2.56	-0.04 [-0.16, 0.07]
NFBC86	1685	2769	9.45	0.02 [-0.04, 0.08]
NINFEA	3111	190	1	0.07 [-0.11, 0.26]
Raine	415	981	2.27	0.02 [-0.10, 0.15]
RHEA	286	144	0.56	0.11 [-0.14, 0.36]
SWS	654	216	1.53	0.22 [0.07, 0.37]
RE Model (I^2 = 91.7)				0.14 [0.07, 0.22]
**8-13 years**				
ABCD	1961	699	4.55	0.75 [0.65, 0.84]
ALSPAC	1348	1421	3.94	0.21 [0.11, 0.32]
BiB	1065	2493	4.09	0.03 [-0.07, 0.13]
CHOP	221	148	0.65	0.47 [0.21, 0.72]
DNBC	22362	9110	59.94	0.25 [0.22, 0.27]
EDEN	586	26	0.23	0.41 [-0.01, 0.83]
ELFE	2706	54	0.48	0.13 [-0.17, 0.42]
GECKO	769	685	3.31	0.33 [0.22, 0.44]
GENR	2658	399	3.25	0.78 [0.67, 0.89]
HGS	1204	542	2.74	0.05 [-0.07, 0.18]
INMA	344	252	1	0.27 [0.07, 0.47]
MoBa	23242	384	3.23	0.19 [0.08, 0.30]
NFBC66	249	6004	2.66	0.04 [-0.08, 0.17]
NFBC86	1159	1819	6.53	0.08 [0.00, 0.16]
NINFEA	687	43	0.28	0.08 [-0.30, 0.46]
Raine	386	929	2.21	0.02 [-0.11, 0.16]
RHEA	129	38	0.2	-0.03 [-0.49, 0.42]
SWS	387	114	0.7	0.50 [0.26, 0.74]
RE Model (I^2 = 94.6)				0.26 [0.14, 0.37]
**14-17 years**				
ALSPAC	1201	1001	18.61	0.23 [0.14, 0.32]
DNBC	2927	1481	33.12	0.28 [0.21, 0.35]
NFBC66	250	5807	10.17	0.08 [-0.05, 0.20]
NFBC86	1434	2166	31.69	0.14 [0.07, 0.21]
Raine	315	733	6.4	0.16 [0.01, 0.32]
RE Model (I^2 = 58.8)				0.19 [0.12, 0.25]

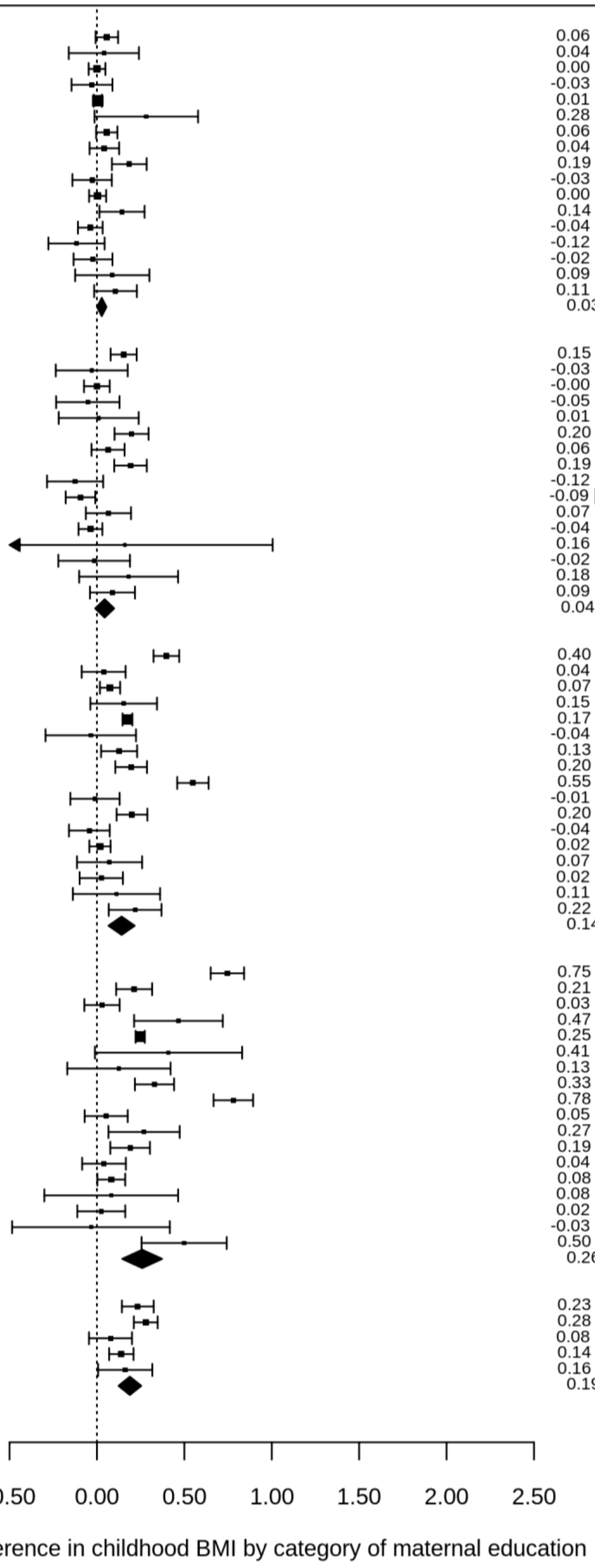

Note: models adjusted for child sex and exact age at measurement.

Web Figure 4: Associations between Normalised Difference Vegetation Index and child BMI z-scores using 2-stage IPD meta-analysis

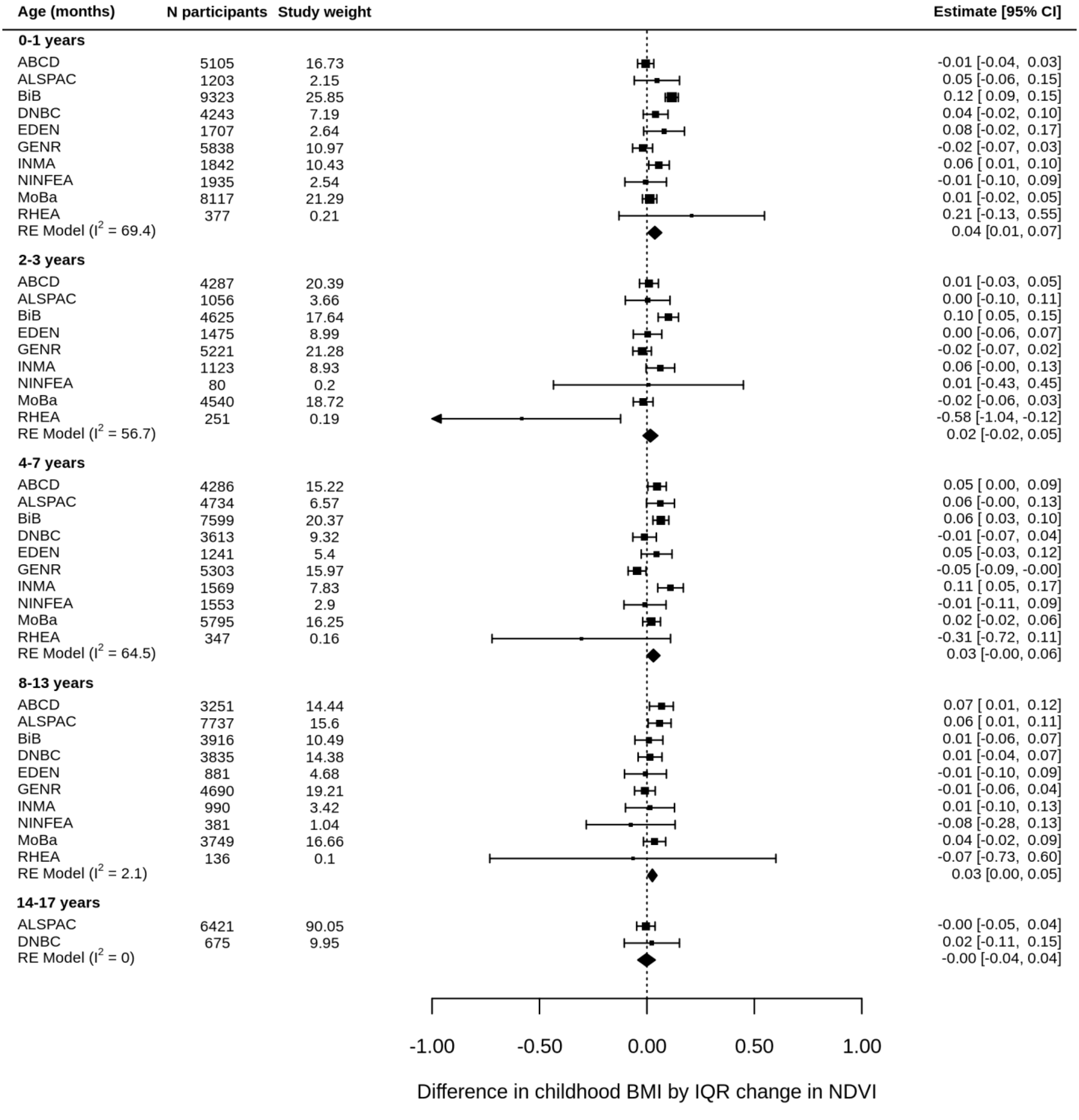

Note: models adjusted for child sex, exact age at measurement, maternal education, parity and area deprivation.

Web Figure 5: Associations between gestational diabetes and child BMI z-scores using 2-stage IPD meta-analysis

Cohort	N participants: Unexposed	N participants: Exposed	Study weight	Estimate [95% CI]
**0-1 years**				
ABCD	4883	64	2.86	0.05 [-0.18, 0.28]
ALSPAC	1063	<5	0.11	1.01 [-0.16, 2.18]
BiB	3729	333	11.61	-0.28 [-0.40, -0.17]
DNBC	49839	436	13.79	-0.05 [-0.16, 0.05]
EDEN	1599	112	1.9	-0.03 [-0.32, 0.25]
ELFE	15388	1149	35.01	0.04 [-0.03, 0.10]
GECKO	2140	56	2.64	0.39 [0.15, 0.63]
GENR	5011	31	0.99	0.35 [-0.04, 0.75]
INMA	1688	78	3.62	0.17 [-0.03, 0.38]
MoBa	78524	349	13.42	-0.03 [-0.14, 0.07]
NINFEA	4988	438	8.83	-0.02 [-0.16, 0.11]
Raine	2040	46	1.81	-0.17 [-0.46, 0.12]
RHEA	750	71	2.04	0.33 [0.06, 0.61]
SWS	2759	33	1.36	0.04 [-0.29, 0.38]
RE Model (I^2 = 77.6)				0.04 [-0.06, 0.14]
**2-3 years**				
ABCD	4118	53	4.35	0.23 [-0.04, 0.51]
ALSPAC	943	<5	0.29	0.39 [-0.66, 1.44]
BiB	1933	179	11.35	-0.10 [-0.27, 0.07]
EDEN	1384	98	8.05	0.04 [-0.16, 0.24]
ELFE	9327	683	45.53	0.04 [-0.05, 0.12]
GECKO	1771	44	4.14	0.04 [-0.24, 0.32]
GENR	4403	29	2.46	0.24 [-0.12, 0.60]
INMA	1020	54	4.21	-0.00 [-0.28, 0.27]
MoBa	42507	180	12.7	0.01 [-0.15, 0.17]
NINFEA	160	27	1.31	-0.50 [-0.99, -0.00]
Raine	559	10	0.85	0.12 [-0.49, 0.73]
RHEA	529	47	2.19	0.04 [-0.34, 0.42]
SWS	2519	30	2.57	0.05 [-0.30, 0.40]
RE Model (I^2 = 0)				0.02 [-0.03, 0.08]
**4-7 years**				
ABCD	4108	57	3.38	0.34 [0.09, 0.59]
ALSPAC	4430	15	0.52	-0.31 [-0.96, 0.33]
BiB	3022	273	11.62	-0.05 [-0.19, 0.08]
DNBC	37757	325	14.02	0.11 [-0.02, 0.23]
EDEN	1161	88	5.08	0.15 [-0.05, 0.36]
ELFE	8876	613	30.44	-0.06 [-0.14, 0.03]
GECKO	1889	46	3.13	0.08 [-0.18, 0.35]
GENR	4500	44	2.63	0.09 [-0.20, 0.37]
INMA	1436	71	3.44	-0.01 [-0.26, 0.24]
MoBa	46212	204	9.33	0.15 [-0.00, 0.30]
NINFEA	3872	330	10.69	-0.09 [-0.23, 0.06]
Raine	1845	42	2.06	0.26 [-0.06, 0.58]
RHEA	676	66	2.28	-0.16 [-0.47, 0.14]
SWS	2023	23	1.39	0.29 [-0.11, 0.68]
RE Model (I^2 = 44.5)				0.05 [-0.02, 0.12]
**8-13 years**				
ABCD	3133	45	4.5	0.36 [0.06, 0.67]
ALSPAC	6823	27	1.72	-0.13 [-0.63, 0.36]
BiB	1446	141	7.62	0.08 [-0.16, 0.31]
DNBC	38655	305	30.15	0.20 [0.08, 0.31]
EDEN	829	55	5.04	0.12 [-0.16, 0.41]
ELFE	2949	190	17.07	0.04 [-0.11, 0.20]
GECKO	1797	44	4.35	0.13 [-0.18, 0.44]
GENR	3990	41	4.06	0.33 [0.01, 0.65]
INMA	913	45	3.11	0.06 [-0.31, 0.43]
MoBa	31174	138	12.36	0.27 [0.09, 0.46]
NINFEA	927	53	3.75	0.17 [-0.17, 0.50]
Raine	1752	35	2.95	0.39 [0.01, 0.77]
RHEA	269	26	1.83	0.36 [-0.12, 0.83]
SWS	1153	17	1.47	0.24 [-0.29, 0.77]
RE Model (I^2 = 0)				0.18 [0.11, 0.24]
**14-17 years**				
ALSPAC	5364	22	24.58	-0.10 [-0.53, 0.33]
DNBC	3057	17	47.66	0.23 [-0.08, 0.54]
Raine	1312	26	27.76	0.21 [-0.20, 0.61]
RE Model (I^2 = 0)				0.14 [-0.07, 0.36]

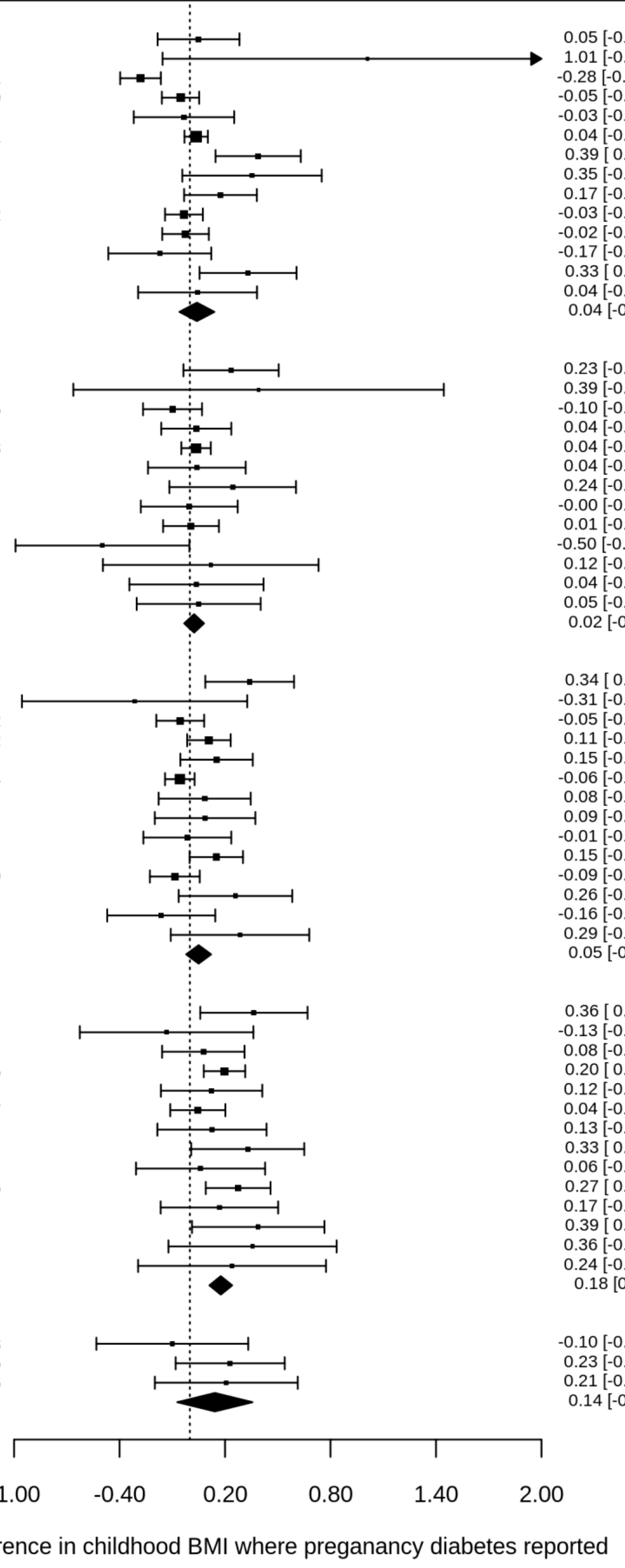

Note: models adjusted for child sex, exact age at measurement, maternal education, parity and pre-pregnancy BMI.

References

1. van Eijsden M, Vrijkotte TG, Gemke RJ, van der Wal MF. Cohort profile: the Amsterdam Born Children and their Development (ABCD) study. Int J Epidemiol. 2011;40(5):1176-86. doi:10.1093/ije/dyq128
2. Boyd A, Golding J, Macleod J, et al. Cohort Profile: the 'children of the 90s'--the index offspring of the Avon Longitudinal Study of Parents and Children. Int J Epidemiol. 2013;42(1):111-27. doi:10.1093/ije/dys064
3. Fraser A, Macdonald-Wallis C, Tilling K, et al. Cohort Profile: the Avon Longitudinal Study of Parents and Children: ALSPAC mothers cohort. Int J Epidemiol. 2013;42(1):97-110. doi:10.1093/ije/dys066
